# Five‐Minute Apgar Scores and Its Prognostic Value for Mortality and Severe Morbidity in Very Preterm Infants: A Multinational Cohort Study

**DOI:** 10.1111/1471-0528.18291

**Published:** 2025-07-15

**Authors:** Harald Ehrhardt, Soodabeh Behboodi, Rolf F. Maier, Adrien M. Aubert, Ulrika Åden, Elizabeth S. Draper, Anna Gudmundsdottir, Veronica Siljehav, Heili Varendi, Tom Weber, Michael Zemlin, Jennifer Zeitlin, the EPICE/SHIPS Research Group, J. Lebeer, P. Van Reempts, E. Bruneel, E. Cloet, A. Oostra, E. Ortibus, I. Sarrechia, K. Boerch, L. Huusom, P. Pedersen, T. Weber, A. Hasselager, L. Toome, H. Varendi, M. Männamaa, P. Y. Ancel, A. Burguet, P. H. Jarreau, V. Pierrat, P. Truffert, R. F. Maier, M. Zemlin, B. Misselwitz, S. Schmidt, L. Wohlers, M. Cuttini, D. Di Lallo, G. Ancora, D. Baronciani, V. Carnielli, I. Croci, G. Faldella, F. Ferrari, F. Franco, G. Gargano, A. van Heijst, C. Koopman‐Esseboom, J. Gadzinowski, J. Mazela, A. Montgomery, T. Pikuła, H. Barros, R. Costa, L. Mendes Graça, M. do Céu Machado, C. Rodrigues, T. Rodrigues, U. Aden, A. K. Edstedt Bonamy, M. Norman, E. S. Draper, E. M. Boyle, A. Fenton, S. J. Johnson, B. N. Manktelow, D. W. A. Milligan, S. Mader, N. Thiele, J. Walz, S. Petrou, J. Zeitlin, M. Bonet, C. Bonnet, R. El Raffei, A. Piedvache, A. V. Seppanen, A. M. Aubert

**Affiliations:** ^1^ Division of Neonatology and Pediatric Intensive Care Medicine, Department of Pediatrics and Adolescent Medicine University Medical Center Ulm Ulm Germany; ^2^ Centre for Research in Epidemiology and StatisticS (CRESS), Obstetrical, Perinatal and Pediatric Lifecourse Epidemiology, OPPaLE Université Paris Cité and Université Sorbonne Paris Nord, Inserm, INRAE Paris France; ^3^ Children's Hospital, University Hospital Philipps University Marburg Marburg Germany; ^4^ Department of Women's and Children's Health Karolinska Institutet Stockholm Sweden; ^5^ Department of Health Sciences University of Leicester Leicester UK; ^6^ University of Tartu Tartu University Hospital Tartu Estonia; ^7^ University of Copenhagen Copenhagen Denmark; ^8^ Hospital for General Pediatrics and Neonatology Saarland University Medical Center Homburg Germany

**Keywords:** Apgar score, bronchopulmonary dysplasia, intraventricular haemorrhage, mortality, preterm infant, prognostic factor, retinopathy of prematurity

## Abstract

**Objective:**

To examine associations between a 5‐min Apgar score < 7 and severe neonatal outcomes in very preterm (VPT) infants and how results are impacted by variations in assigning Apgar scores within an international context.

**Design:**

Prospective observational population‐based cohort study.

**Setting:**

Eleven structurally and organisationally diverse countries across Europe.

**Population:**

In total, 7900 liveborn VPT infants from the EPICE‐SHIPS study.

**Methods:**

Descriptive statistics, logistic regression, modified Poisson regression.

**Main Outcome Measures:**

Associations between 5‐min Apgar scores < 7 and adverse neonatal outcomes were estimated with adjustments for perinatal characteristics. We tested for interactions by country‐level prevalence of an Apgar score < 7, grouped into low (14%–16%), medium (19%–22%) and high (28%–40%).

**Results:**

20.2% of infants had 5‐min Apgar score < 7 with rates of 14%–40% across countries. A score < 7 increased risks of in‐hospital mortality, intraventricular haemorrhage (IVH), cystic periventricular leukomalacia (cPVL), retinopathy of prematurity (ROP), bronchopulmonary dysplasia (BPD) and length of hospital stay (LHS), but not necrotising enterocolitis or late‐onset infection (LOI). No interactions with country group were detected for mortality, cPVL and ROP, while associations with IVH, BPD and LHS were restricted to countries with lower prevalence of scores < 7.

**Conclusions:**

Significant differences exist in the prevalence of low Apgar scores across countries. Their interactions with adverse outcomes demand caution when using the Apgar score in prognostic models for clinical care and research without local validation. More broadly, our findings emphasise the importance of accounting for country‐specific effects in clinical assessment scores.

## 1 Introduction

The Apgar score was originally developed to standardise the assessment of term‐born infants' clinical condition at 1 min of life. However, accumulating research shows that the judgement at 5 and 10 min has better predictive value for in‐hospital mortality and severe neurologic and non‐neurologic morbidities in term‐born infants [1, 2, 3, 4]. In very preterm (VPT) infants < 32 weeks' gestation, low 5‐min Apgar scores have been consistently associated with increased risk of mortality, but results on associations with morbidity are contradictory [1, 5, 6]. The authors from a multinational research collaboration concluded that low Apgar scores were associated with increased risk for severe brain injury in preterm infants 24–28 weeks but not in a graded manner [7]. The longer‐term prognostic value was called into question in extremely preterm infants < 28 weeks as no correlation was found between low Apgar scores and 5‐year cognitive or motor abilities [8].

One major concern is the variation in Apgar scoring [9, 10, 11]. In the general population, marked differences in the distribution of Apgar scores were observed between 23 European countries in the Euro‐Peristat network [12]. This variability may be more acute for VPT infants in the absence of clear guidance for scoring in this population and with their clinical condition, characterised by reduced signs of vitality in the first minutes of life and worse responses to stabilisation [5, 7].

Key aims of the present study were to describe variations in 5‐min Apgar scores < 7 among VPT infants across European countries, to assess associations with adverse neonatal outcomes and to test whether these associations differ by country‐level variations in low Apgar score prevalence.

## 2 Methods

### 2.1 Study Design and Ethics Approval

The EPICE‐SHIPS cohort is a prospective population‐based study of all stillbirths and live births from 22 + 0 to 31 + 6 weeks' gestation across 19 regions in 11 European countries. Countries (regions) were Belgium (Flanders), Denmark (Eastern), Estonia (entire country), France (Burgundy, Ile‐de‐France, Northern), Germany (Hesse, Saarland), Italy (Emilia‐Romagna, Lazio, Marche), the Netherlands (Central and Eastern), Poland (Wielkopolska), Portugal (Lisbon and Northern), Sweden (Stockholm) and the United Kingdom (East Midlands, Northern and Yorkshire and Humber) [13]. Data were collected between April 2011 and September 2012 over a 12‐month period, except in the French regions where data were collected during a 6‐month interval. Ethical approvals were obtained from regional and/or hospital ethics committees. The study was also approved by the French Advisory Committee on Use of Health Data in Medical Research (CCTIRS No. 13.020) and the French National Commission for Data Protection and Liberties (CNIL No. DR‐2013‐194). Parental consent was required for follow‐up, but waivers allowed inclusion of all births minimising refusals (*N* = 20/8282, 0.2%), except for the French regions where parental consent was required for perinatal data (*N* = 140/2047, 6.8%) This study adheres to the STROBE reporting guideline for observational studies.

### 2.2 Study Population

The study included all 7900 liveborn VPT infants in the cohort.

### 2.3 Data Collection and Outcome Definitions

Baseline characteristics and outcome data were retrieved from patient records using a standardised questionnaire with pretested definitions by trained personnel in the maternity and neonatal units. Data were collected until discharge home or into long‐term care. Preterm premature rupture of membranes (PPROM) was defined as > 12 h before the onset of labour. Inborn status referred to birth in a unit with an appropriate level of care, as defined by national legislation. Caesarean sections were separated into those before the onset of labour and during labour. Administration of antenatal steroids (ANS) was counted when at least one dose was given before delivery.

Serious congenital anomalies were based on the Eurocat definitions and lethality [14]. Gestational age was based on obstetric assessment and information on the last menstrual period and routine ultrasound measures. Small for gestational age (SGA) status was categorised into birthweight < 3rd, 3rd to 9th and ≥ 10th percentile using intrauterine charts as developed for the cohort [15]. Adverse neonatal outcomes included: in‐hospital mortality separated into labour ward death and mortality during the stay in the neonatal intensive care unit (NICU), severe intraventricular haemorrhage (IVH) >grade 2, necrotising enterocolitis (NEC) requiring surgical therapy or peritoneal drainage, late onset infection (LOI) commencing after the first 72 h of life, cystic periventricular leukomalacia (cPVL), retinopathy of prematurity (ROP) ≥stage 3, moderate or severe bronchopulmonary dysplasia (BPD) according to the 2001 NICHD consensus definition [16, 17, 18]. We also investigated prolonged length of hospital stay (LHS) among infants surviving to discharge, categorised as prolonged when gestational age at discharge was in the top quintile of the country‐specific gestational age–standardised postmenstrual age at discharge [19].

### 2.4 Exposure

Our exposure was the 5‐min Apgar score in conformity with previous studies on this topic where a value < 7 was set as cut‐off for an adverse outcome [1, 3, 5, 7].

### 2.5 Missing Data

Data on Apgar were missing for 6.4% of the sample. For maternal, perinatal and management covariables, the proportions of missing data were low, with the highest proportions for maternal country of birth (3.8%). For the outcome variables, missing data were also infrequent (< 4%).

### 2.6 Statistical Analysis

We compared infants with and without Apgar scores to assess any possible biases. Then, we described the distribution of maternal, perinatal and management characteristics and the prevalence of a 5‐min Apgar < 7 by these characteristics. We also assessed the unadjusted and adjusted prevalence of a 5‐min Apgar < 7 by country. Adjusted prevalence was computed by running a logistic model including sociodemographic, perinatal characteristics and management variables (maternal age, maternal country of birth, gestational age, SGA, singleton/multiple pregnancy, maternal parity, PPROM, preeclampsia/eclampsia/HELLP syndrome) and predicting the value for each country based on the case‐mix in the full sample. We created three data‐driven groups of countries based on the prevalence of low 5‐min Apgar scores that best separated different score rates and verified their consistency after adjustment.

Relative risks (RR) of experiencing adverse neonatal outcomes associated with low 5‐min Apgar scores were estimated using modified Poisson regression with several sequential models: adjusted (1) for country, (2) for country and perinatal characteristics (gestational age, SGA, multiple birth, sex, PPROM, parity, preeclampsia/eclampsia/HELLP) and (3) for country, perinatal characteristics and management variables (ANS, inborn status, composite variable of onset of labour and mode of delivery) [8, 17, 20]. To explore whether associations between low 5‐min Apgar and adverse outcomes differed in relation to the country‐level prevalence of low Apgar, we introduced country group as an interaction term into our final models to compute the group‐specific effect of low Apgar. Although missing data prevalence was low in our sample, we carried out multiple imputation by chained equations (MICE, *m* = 20) to impute missing data for covariables used in all adjusted analyses in order to retain the full sample [21].

We carried out several sensitivity analyses of our final models first providing a complete‐case analyses without imputed covariables, then excluding infants born < 24 + 0 weeks' gestation and additionally infants with severe congenital malformation, as these infants have a predisposition for poor postnatal adaptation in the delivery room that can affect 5‐min Apgar score values and outcomes [22, 23]. All analyses were carried out using STATA software version 15.0 (StataCorp, College Station, TX, USA).

## 3 Results

### 3.1 Variable Distribution by 5‐Min Apgar Score

From the total population of 7900 VPT infants, 5‐min Apgar scores were available in 7396 infants (93.6%). Apgar scores were more often missing in outborn infants, at lower gestational ages, for vaginal deliveries and in cases with no ANS (Table S1). The median [interquartile range] of the 5‐min Apgar score was 8 [8, 9, 10]. One‐fifth (1494/7396, 20.2%) of VPT infants had 5‐min Apgar scores < 7. Five‐minute Apgar scores < 7 were more common in infants of mothers aged < 25 years, who were foreign born and of African origin. Infants with low 5‐min Apgar were more often singletons, of lower gestational age, without ANS, outborn, born by vaginal delivery, with severe congenital malformations, but less likely to be from deliveries complicated by preeclampsia/eclampsia/HELLP syndrome and to be SGA (Table 1).

**TABLE 1:** Maternal and perinatal characteristics of the overall sample and of infants with 5‐min Apgar ≥ 7 and < 7.

	Sample characteristics		Sample characteristics by 5‐min Apgar			
	All births (*N* = 7396)		Apgar ≥ 7 (*N* = 5902)		Apgar < 7 (*N* = 1494)	
	*N*	%	*N*	%	*N*	%
Maternal age at childbirth, years						
< 25	1250	17.0	954	16.2	296	19.9
25–34	4192	56.9	3376	57.4	816	54.8
≥ 35	1927	26.2	1551	26.4	376	25.3
Maternal parity						
Primiparous	4125	56.3	3325	56.9	800	54.0
Multiparous	3204	43.7	2522	43.1	682	46.0
Country of birth						
Born in country	5382	75.9	4361	76.5	1021	73.5
Born in another European country	443	6.2	372	6.5	71	5.1
Born outside of Europe	1266	17.9	969	17.0	297	21.4
Continental country classification						
Africa	755	10.6	558	9.8	197	14.2
America	133	1.9	110	1.9	23	1.7
Asia^a^	380	5.4	303	5.3	77	5.5
Europe	5825	82.1	4733	83.0	1092	78.6
Type of pregnancy						
Singleton	5083	68.7	3973	67.3	1110	74.3
Multiple	2312	31.3	1928	32.7	384	25.7
Preeclampsia, eclampsia or HELLP syndrome						
No	6151	84.7	4859	83.7	1292	88.7
Yes	1110	15.3	945	16.3	165	11.3
PPROM > 12 h						
No	5451	75.1	4377	75.4	1074	74.0
Yes	1809	24.9	1431	24.6	378	26.0
Inborn						
No	769	10.7	571	9.7	198	15.2
Yes	6417	89.3	5314	90.3	1103	84.8
Any antenatal steroids						
No	927	12.6	568	9.7	359	24.3
Yes	6405	87.4	5286	90.3	1119	75.7
Mode of delivery						
Vaginal birth	2269	31.0	1696	29.0	573	39.0
Instrumental vaginal birth	170	2.3	127	2.2	43	2.9
Prelabour caesarean	3037	41.5	2531	43.3	506	34.4
Intrapartum caesarean	1842	25.2	1495	25.6	347	23.6
Sex of the infant						
Male	4012	54.3	3164	53.6	848	56.8
Female	3380	45.7	2735	46.4	645	43.2
Undetermined	1	0.0	1	0.0	0	0.0
Gestational age, completed weeks						
23/24	604	8.2	233	3.9	371	24.8
25	400	5.4	250	4.2	150	10.0
26	548	7.4	399	6.8	149	10.0
27	697	9.4	520	8.8	177	11.8
28	868	11.7	698	11.8	170	11.4
29	1000	13.5	853	14.5	147	9.8
30	1439	19.5	1282	21.7	157	10.5
31	1840	24.9	1667	28.2	173	11.6
SGA centiles, using intrauterine charts						
< 3	1535	20.8	1281	21.7	254	17.0
3–10	844	11.4	683	11.6	161	10.8
> 10	5013	67.8	3935	66.7	1078	72.2
Severe congenital malformation						
No	7280	98.4	5828	98.8	1452	97.2
Yes	115	1.6	73	1.2	42	2.8

*Note:* Data are given as absolute numbers and percentages of rows separated by the 5‐min Apgar score. The total sample size differs for some variables due to missing observations. *p*‐values were calculated using chi‐squared tests.

Abbreviations: ANS, antenatal steroids; C‐section, caesarean section; PPROM, preterm premature rupture of membranes; SGA, small for gestational age.

^a^ Includes two women from the Pacific islands.

### 3.2 Differences Between Countries in the Prevalence of a Low 5‐Min Apgar Score

**FIGURE 1:**
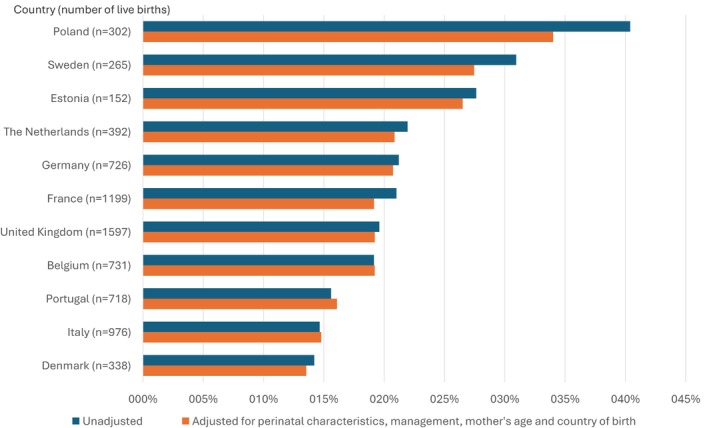
Distribution of unadjusted and adjusted prevalence of low Apgar score at 5‐min < 7 across European countries in very preterm infants, all live births. Calculated were the relative frequencies of a 5‐min Apgar score < 7 across the 11 European countries on country‐level. Adjustment was executed running a logistic model including variables associated with low Apgar scores (gestational age, SGA, type of pregnancy, maternal parity, PPROM, preeclampsia, eclampsia or HELLP syndrome) and predicting the value for each country based on the case‐mix in the full sample and using multiple imputation by chained equations (MICE) to impute missing data for covariables. Frequencies are displayed as mean and range in descending order.

The prevalence of a 5‐min Apgar score < 7 varied by country. It was below 16% in Denmark, Italy and Portugal (classified in the low group), between 19% and 22% in Belgium, the United Kingdom, France, Germany and the Netherlands (medium group) and 28% or above in Estonia, Sweden and Portugal (high group) (Figure 1). Country groups differed in their characteristics, with the high group having more multiparous women, native‐born mothers, extreme preterm infants and fewer growth‐restricted infants (Table S2). Adjustment for population characteristics modified some individual country rates, but country groupings remained the same.

### 3.3 Association of the 5‐Min Apgar Score With Adverse Neonatal Outcomes

Among infants with a 5‐min Apgar score < 7, 35.7% (534/1494) died while this percentage was 6.9% (406/5902) with Apgar scores ≥ 7 (Table 2). The risk of death in the labour ward was strongly increased with a 5‐min Apgar score < 7 compared to infants with an Apgar score ≥ 7, but NICU mortality remained significantly higher with a 5‐min Apgar score < 7. In unadjusted comparisons, the incidence of all adverse neonatal outcomes was higher for infants with a 5‐min Apgar score < 7 (Table 2).

**TABLE 2:** Adverse neonatal outcomes overall and separated by 5‐min Apgar ≥ 7 and < 7.

Adverse neonatal outcomes	All births			Apgar ≥ 7			Apgar < 7			*p*
	*N*	*n*	%	*N*	*n*	%	*N*	*n*	%	
In‐hospital death^1^	7396	940	12.7	5902	406	6.9	1494	534	35.7	< 0.001
Death in the labour ward	7396	263	3.6	5902	15	0.3	1494	248	16.6	< 0.001
Death after NICU admission	7133	677	9.5	5887	391	6.6	1246	286	23.0	< 0.001
IVH > grade 2^2^	7037	457	6.5	5807	274	4.7	1230	183	14.9	< 0.001
Surgical NEC^3^	7199	174	2.4	5897	123	2.1	1302	51	3.9	< 0.001
Late onset infection^4^	7042	1971	28.0	5764	1553	26.9	1278	418	32.7	< 0.001
cPVL^5^	7023	235	3.3	5813	170	2.9	1210	65	5.4	< 0.001
ROP ≥ stage 3^6^	7072	235	3.3	5798	147	2.5	1274	88	6.9	< 0.001
BPD (moderate/severe)^7^	6417	956	14.9	5451	686	12.6	966	270	28.0	< 0.001
Length of hospital stay (top quintile)^8^	6188	1137	18.4	5301	921	17.4	887	216	24.4	< 0.001

*Note:* Data are given as absolute numbers and percentages of rows separated by the 5‐min Apgar score. The total number of patients differs for missing values for individual covariables due to death before diagnosis or missing data (^1^ *n* = 2; ^2^ *n* = 359; ^3^ *n* = 197; ^4^ *n* = 354; ^5^ *n* = 373; ^6^ *n* = 324; ^7^ *n* = 979; ^8^ *n* = 1208). *p*‐values were calculated using chi squared tests.

Abbreviations: BPD, bronchopulmonary dysplasia; cPVL, cystic periventricular leukomalacia; IVH, intraventricular haemorrhage; NEC, necrotising enterocolitis; NICU, neonatal intensive care unit; ROP, retinopathy of prematurity.

**FIGURE 2:**
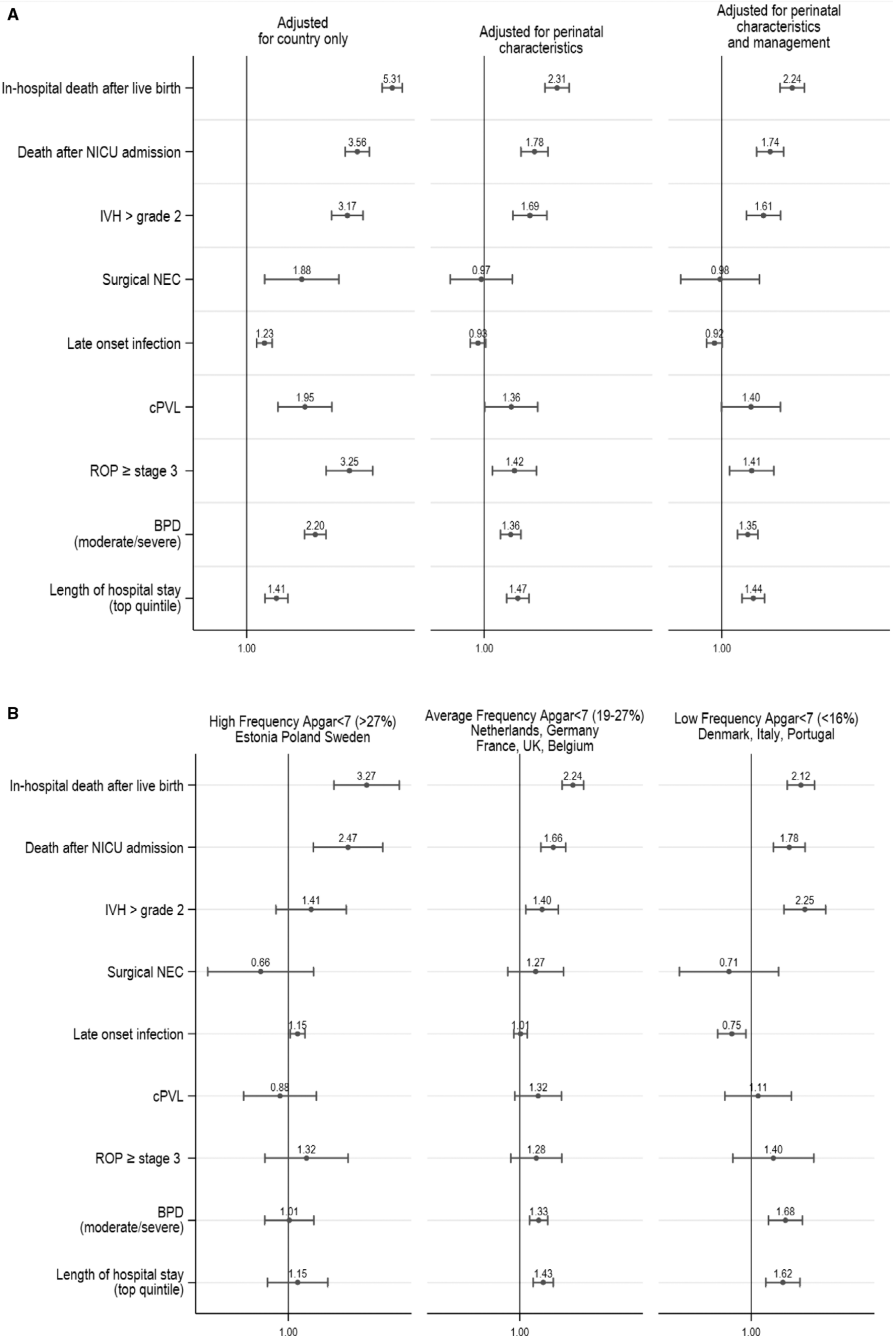
Relative risks of adverse neonatal outcome associated with an Apgar score < 7 at 5 min in very preterm infants: all live births and segregated by country group, classified by low Apgar prevalence. (A) Data were analysed using modified Poisson regression without adjustments except for country, with additional adjustment for baseline characteristics (gestational age, small for gestational age, multiple birth, sex, PPROM, parity, preeclampsia, eclampsia or HELLP syndrome), and plus adjustment for the perinatal management (antenatal steroids, inborn and composite variable of onset of labour and mode of delivery), fully adjusted models using multiple imputation by chained equations (MICE) to impute missing data for covariables. (B) Analyses from (A) adjusted for baseline characteristic (gestational age, small for gestational age, multiple birth, sex, PPROM, parity, preeclampsia, eclampsia or HELLP syndrome) and perinatal management (antenatal steroids, inborn and composite variable of onset of labour and mode of delivery) were conducted using an interaction term to derive coefficients by country group: higher (Poland, Sweden, Estonia), average (the Netherlands, Germany, France, the UK, Belgium) and lower (Portugal, Italy, Denmark) frequencies of a 5‐min Apgar score < 7. Proportion of 5‐min Apgar score < 7 in countries: Poland (40.4%), Sweden (30.9%), Estonia (27.8%), Netherlands (21.9%), Germany (21.2%), France (21.0%), United Kingdom (19.6%), Belgium (19.1%), Portugal (15.6%), Italy (14.6%), Denmark (14.2%). Presented are relative risks and 95% confidence intervals. BPD, bronchopulmonary dysplasia; cPVL, cystic periventricular leukomalacia; IVH, intraventricular haemorrhage; NEC, necrotizing enterocolitis; NICU, neonatal intensive care unit; ROP, retinopathy of prematurity.

Figure 2A presents the sequential models associating low 5‐min Apgar with adverse neonatal outcomes. After adjusting for country only, the associations were significant for all neonatal outcomes. The higher probability of in‐hospital mortality in models adjusted for country (RR = 5.31; 95% confidence interval (CI): 4.73–5.97) persisted after adjustment for perinatal characteristics and clinical management (RR = 2.24; 95% CI: 1.95–2.58). Similar results were observed for deaths after NICU admission (RR = 1.74; 95% CI: 1.49–2.04). Associations with a 5‐min Apgar score < 7 were found for IVH (RR = 1.61; 95% CI: 1.33–1.96), ROP (RR = 1.41; 95% CI: 1.09–1.82), cPVL (RR = 1.40; 95% CI: 1.00–1.96), BPD (RR = 1.35; 95% CI: 1.20–1.51) and LHS (RR = 1.44; 95% CI: 1.26–1.63), but not for NEC (RR = 0.98; 95% CI: 0.63–1.54) and LOI (RR = 0.92; 95% CI: 0.84–1.01).

Sensitivity analysis, restricting the model to the complete‐case sample (Figure S1), to infants ≥ 24 weeks (Figure S2) and excluding infants with severe congenital anomalies (Figure S3) did not change the results.

### 3.4 Impact of Variations in 5‐Min Apgar Scores Between the Countries

After complete adjustment for baseline characteristics and perinatal management, the RR for in‐hospital death after live birth, for deaths in the labour ward and after transfer to the NICU was present regardless of the country category while other adverse outcomes remained unassociated with a low 5‐min Apgar score in all country groups (Figure 2B, Figures S4–S11). In contrast, there were stronger associations in the group of countries with a lower prevalence of 5‐min Apgar < 7 for IVH (test of interaction, *p* = 0.04), BPD (*p* = 0.09) and LHS (*p* = 0.43).

## 4 Discussion

### 4.1 Main Findings

In a multinational cohort of VPT infants using data abstracted following a common, standardised protocol, we provide novel results on the association of 5‐min Apgar score < 7 and multiple severe outcomes [24]. Three findings advance the scientific knowledge about the prognostic value of the 5‐min Apgar score in this high‐risk population. First, the proportion of VPT infants with 5‐min Apgar scores < 7 varies largely between European countries. Second, there is a clinically relevant association of low 5‐min Apgar scores with most adverse neonatal outcomes. This is reassuring as the Apgar score is the first clinical assessment to judge the vitality of VPT infants after birth. The importance arises from the fact that the score guides clinical treatment decisions for stabilisation measures and resuscitation worldwide, although concerns of its suitability have been raised by researchers and leading medical societies [25, 26]. Lastly, the variations in strength of associations between adverse outcomes and low 5‐min Apgar score by country suggest the need for context‐specific validation of the Apgar score and risk thresholds. While mortality was not impacted by country variations in 5‐min Apgar scores, this was present for severe IVH. The latter finding is of relevance for the long‐term outcome of VPT infants, as severe IVH constitutes one of the most devastating acute morbidities with high impact on the psychomotor outcome.

### 4.2 Results in the Context of Other Evidence

Our findings, showing high variability in 5‐min Apgar scores across countries, are in line with data on the Apgar score distribution in general populations from the Euro‐Peristat network [12]. However, the association of a low 5‐min Apgar score and acute severe outcomes has not previously been investigated on a multi‐national level in the VPT population except for the iNeo research collaborative of 11 high‐income countries. Our results advance this knowledge by assessing multiple outcomes relevant for the outcome of VPT births and estimating differential effects of Apgar on outcomes in country groups with different Apgar distributions.

Similarly to the iNeo study, we find that risk ratios were higher for mortality than for severe IVH and/or cPVL [7] and further show that the association of the 5‐min Apgar score with multiple adverse neonatal outcomes was not uniform. For in‐hospital mortality, we confirm the association that was established in a population‐based analysis from the Swedish medical birth registry [5]. The authors described a decrease in associations at lower gestational ages that we were not able to address due to the smaller sample size. However, we were able to differentiate between deaths in the delivery room, where the association was extremely high, and those after NICU admission. We documented associations for all adverse neonatal outcomes except NEC and LOI, which constitute infection‐driven morbidities. An explanation for this disparity could be the high impact of clinical management on NEC and LOI, while for the other morbidities, perinatal characteristics and management, more likely to be reflected in the 5‐min Apgar score, are of more relevance [27, 28]. Support for this hypothesis was provided in recent results from the Vermont Oxford Network [29].

Our results showing an association with severe neonatal outcomes in infants < 32 weeks' gestation contrast with those from an analysis of the 5‐min Apgar score and 5‐year cognitive and motor outcomes in the EPICE‐SHIPS cohort. This study did not find an association, even with Apgar scores of 0 to 3, in the subpopulation of extremely preterm infants < 28 weeks' gestation [8]. Our current study found an association with severe adverse neonatal outcomes for the total population of VPT infants < 32 weeks' gestation, but it is unlikely that the different populations studied explain the discordant findings. However, a decisive difference between our two studies is the observation period. Although there is a well‐known association between adverse neonatal outcomes and 5‐year psychomotor development, these latter outcomes are impacted by the socioeconomic status of the families and the availability of support measures which may mitigate their long‐term effects [24, 30, 31, 32, 33, 34]. The publications from our and other cohorts document the marked impact of the post‐discharge period and thereby stress the importance of focusing on acute adverse outcomes as well as follow‐up for sustainable improvements of outcomes in VPT infants [7, 8, 24, 30, 35, 36, 37]. Nonetheless, we might have found a difference in longer‐term outcomes if we had investigated the countries where a low 5‐min Apgar score was more strongly associated with neonatal morbidity and this remains an area for further research using larger samples to confirm these country‐specific effects and to detect long‐term consequences on child health and development.

### 4.3 Strengths and Limitations of the Study

The major strength of our study is the prospective and complete data collection from 19 geographically and organisationally diverse regions in 11 European countries allowing analyses on real‐life data and applicability of our results to diverse clinical care settings. We applied step‐wise risk adjustment, including only country to account for the variations in the proportion of low 5‐min Apgar scores, followed by perinatal characteristics and then perinatal management. Additional sensitivity analyses restricting our sample by removing congenital anomalies and infants born before 24 weeks' gestation, confirmed the robustness of our results.

Nevertheless, we acknowledge limitations: Data were collected from medical records and we did not have information about how the 5‐min Apgar score was judged in the centres. Thereby, we cannot describe or control for variations in scoring [9, 10]. It is reassuring that countries that were identified in the low and high prevalence group were similarly low versus high in the European general population [12]. The data were collected in 2011 and 2012 and Apgar judgement and clinical practices have changed since that time [38, 39]. For example, scoring may be affected by changes in the life support algorithm for VPT infants, stricter pulse oximetry monitoring or a gentler respiratory approach. Other factors may relate to evolving oxygen saturation targets, hypothermia prevention strategies and nurse to patient ratios. Furthermore, attitudes towards survival‐focused care at 22 and 23 weeks' gestation have changed in several countries, meaning that our results may not apply to this subgroup in current clinical practice. Further, analysis was limited by the absence of data on intent to resuscitate and postnatal resuscitation. But we undertook sensitivity analyses, excluding these births (< 3% of our sample), and found very similar overall results. Although we had a large sample, it was not sufficient for further inquiry by specific gestational age strata or individual countries. We were also limited in assessing possible interactions with population characteristics or medical practices within our three country groups. We focussed on the 5‐min Apgar score for its higher validity compared to the judgement at 1 min after birth and because it less likely is impacted by postnatal treatment than the 10‐min score [1, 2]. Our study is based on a mostly white European sample; therefore, our findings should be validated on cohorts with populations of diverse origins and races [7, 11]. Further investigation would also be of interest to understand the lower 5‐min Apgar scores in some population groups suggested in our descriptive data.

### 4.4 Interpretation

Early prediction of adverse outcomes remains an unmet need in research on VPT infants and clinical care [40]. Easy‐to‐use bedside tests are required that can guide prompt decisions as well as risk stratification [41, 42]. The 5‐min Apgar score has remained a standard assessment tool for more than 70 years despite the advances in clinical care and medical knowledge. One of the reasons is its easy applicability. Our results and others argue for its suitability to predict adverse neonatal outcomes after VPT birth [41, 42, 43]. However, associations are too weak and variability is too high to base treatment decisions exclusively on the 5‐min Apgar score. Multivariable models, combining the 5‐min Apgar score with additional items including medical interventions after delivery and the new features of artificial intelligence, show promise, as established for other disease entities like sepsis [44, 45, 46]. This will hopefully lead to an easy‐to‐handle tool with high accuracy as established for BPD and ROP risk calculators [47, 48, 49]. Overall, our results encourage initiatives to standardise Apgar scoring within a European guideline and video tutorials.

## 5 Conclusion

Our results indicate that low 5‐min Apgar scores < 7 are associated with an unfavourable short‐term outcome in VPT infants. The variations in Apgar scores between the countries underscore the importance of not only focusing on baseline risks, management and outcomes, but also considering the interaction with country in routine clinical care and research.

## Author Contributions

Jennifer Zeitlin: conceptualised and designed the study, had full access to the study datasets and data analyses and drafted the manuscript. Harald Ehrhardt and Rolf F. Maier: conceptualised and designed the study, revised for important intellectual content and drafted the manuscript. Soodabeh Beeboodhi: had full access to the study datasets and data analyses, revised for important intellectual content and drafted the manuscript. All authors: investigated, interpreted the data, critically revised the manuscript for important intellectual content, approved the final version of the manuscript and were accountable for all aspects of the work in ensuring that questions related to the accuracy or integrity of any part of the work were appropriately investigated and resolved: all authors and all members of the EPICE and SHIPS research group.

## Conflicts of Interest

Harald Ehrhardt reports grants from the German Research Foundation (DFG), grants from the Federal Ministry of Education and Research (BMBF), grants from the von Behring‐Röntgen‐Foundation and grants from the Chiesi Research Foundation outside the submitted work; and Harald Ehrhardt is a member of the European Neonatal Research Consortium of the European Society for Paediatric Research (ESPR) and a member of the European Association of Perinatal Medicine (EAPM) special interest group of preterm delivery. No lectures, presentations, travel or personal reimbursement have been financed by any company. Soodabeh Behboodi has nothing to disclose. Rolf Maier received grants from the European Union's Horizon 2020, Grant/Award Number: 633724 during the conduct of the study. Adrien Aubert has nothing to disclose. Ulrika Åden is President of the Swedish Paediatric Society (unpaid). Elizabeth Draper has nothing to disclose. Anna Gudmundsdottir reports grants from Sällskapet Barnavård, Sweden and grants from Stiftelsen Samariten, Sweden during the conduct of the study. Veronica Siljehav received funding from the Swedish heart‐lung foundation outside of the study and is a board member of the Swedish paediatric cardiology association. Heili Varendi received funding paid to the University of Tartu from the European Union's Seventh Framework Programme (FP7/2007‐2013, No. 259882) and from the European Union's Horizon 2020 research and innovation programme (No. 633724 and No. 733280) during the conduct of the study. Tom Weber received funding from the European Union's Horizon 2020 (Grant/Award Number: 633724) during the conduct of the study. Michael Zemlin received funding paid to the University of Homburg from the European Union's Horizon 2020 research and innovation programme (No. 633724) during the conduct of the study. Jennifer Zeitlin received funding paid to the University of Homburg from the European Union's Horizon 2020 research and innovation programme (No. 633724) during the conduct of the study.

## Supporting information


Data S1:

## Data Availability

Deidentified individual participant data will not be made available as access to datasets of the EPICE and SHIPS cohort is currently not possible for researchers outside the consortium, but the study is part of a H2020 project (RECAP, https://recap‐preterm.eu/) to develop a platform for data sharing. The corresponding author is available for more information.
