# Supplementary material for: Five‐Minute Apgar Scores and Its Prognostic Value for Mortality and Severe Morbidity in Very Preterm Infants: A Multinational Cohort Study
Source: BJOG. 2025 Jul 15;133(4):649–59. doi: 10.1111/1471-0528.18291 (PMC12884215; doi:10.1111/1471-0528.18291)
Supplement: Supplementary file 1 — Data S1: [file BJO-133-649-s001.pdf]

Supplemental Material

Ehrhardt H, Behboodi S, Maier RF, Aubert AM, Åden U, Draper ES, Gudmundsdottir A, Siljehav V, Varendi H, Weber T, Zemlin M, Zeitlin J. **Five-minute Apgar scores and its prognostic value for mortality and severe morbidity in very preterm infants: a multinational cohort study.**

Figure S1. Relative risks of adverse neonatal outcome associated with an Apgar score <7 at five minutes in very preterm birth infants ≥ 24 weeks, all live births with complete case sample.

Figure S2. Relative risks of adverse neonatal outcome associated with an Apgar score <7 at five minutes in very preterm birth infants ≥ 24 weeks, all live births.

Figure S3. Relative risks of adverse neonatal outcome associated with an Apgar score <7 at five minutes in very preterm birth infants ≥ 24 weeks without severe congenital malformation, all live births.

Figure S4. Unadjusted relative risks of adverse neonatal outcome among very preterm infants associated with an Apgar score <7 at five minutes by country group, classified by low Apgar prevalence

Figure S5. Unadjusted relative risks of adverse neonatal outcome among very preterm infants ≥ 24 weeks associated with an Apgar score <7 at five minutes by country group, classified by low Apgar prevalence

Figure S6. Unadjusted relative risks of adverse neonatal outcome among very preterm infants ≥ 24 weeks without severe congenital malformations associated with an Apgar score <7 at five minutes by country group, classified by low Apgar prevalence

Figure S7. Relative risks of adverse neonatal outcome among very preterm infants associated with an Apgar score <7 at five minutes by country group, classified by low Apgar prevalence, adjusted for baseline characteristics

Figure S8. Relative risks of adverse neonatal outcome among very preterm infants ≥ 24 weeks associated with an Apgar score <7 at five minutes by country group, classified by low Apgar prevalence, adjusted for baseline characteristics

Figure S9. Relative risks of adverse neonatal outcome among very preterm infants ≥ 24 weeks without severe congenital malformations associated with an Apgar score <7 at five minutes by country group, classified by low Apgar prevalence, adjusted for baseline characteristics

Figure S10. Relative risks of adverse neonatal outcome among very preterm infants ≥ 24 weeks associated with an Apgar score <7 at five minutes by country group, classified by low Apgar prevalence, adjusted for baseline characteristics and perinatal management

Figure S11. Relative risks of adverse neonatal outcome among very preterm infants ≥ 24 weeks without severe congenital malformations associated with an Apgar score <7 at five minutes by country group, classified by low Apgar prevalence, adjusted for baseline characteristics and perinatal management

Table S1. Maternal and perinatal characteristics of infants with and without Apgar scores

Table S2. Characteristics of infants in the three country Apgar groups

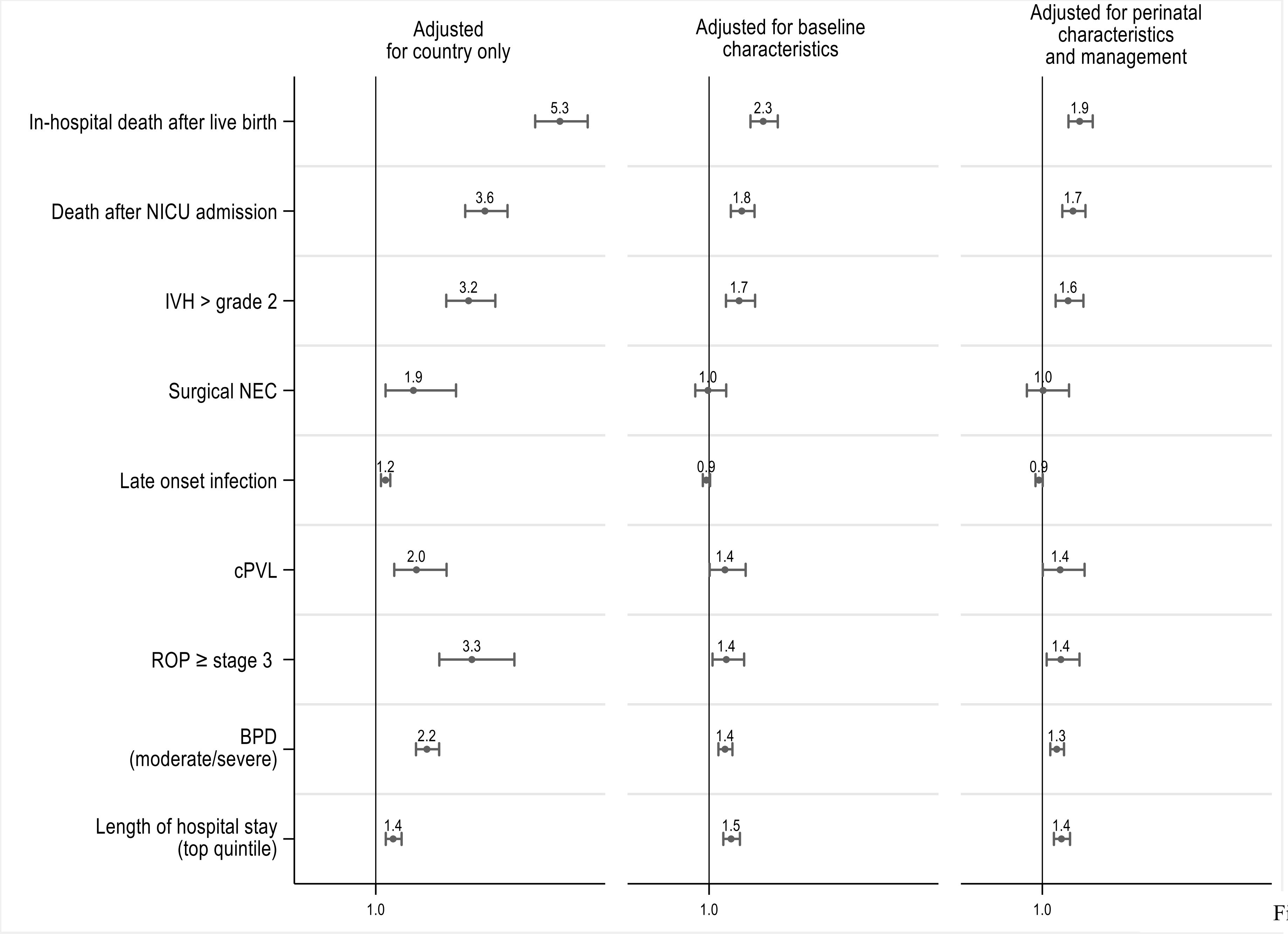
Adjusted
for country only
Adjusted for baseline
characteristics
Adjusted for perinatal
characteristics
and management
In-hospital death after live birth
Death after NICU admission
IVH > grade 2
Surgical NEC
Late onset infection
cPVL
ROP ≥ stage 3
BPD
(moderate/severe)
Length of hospital stay
(top quintile)
5.3
3.6
3.2
1.9
1.2
2.0
3.3
2.2
1.4
2.3
1.8
1.7
1.0
0.9
1.4
1.4
1.4
1.5
1.9
1.7
1.6
1.0
0.9
1.4
1.4
1.3
1.4
1.0
1.0
1.0

Figure S1

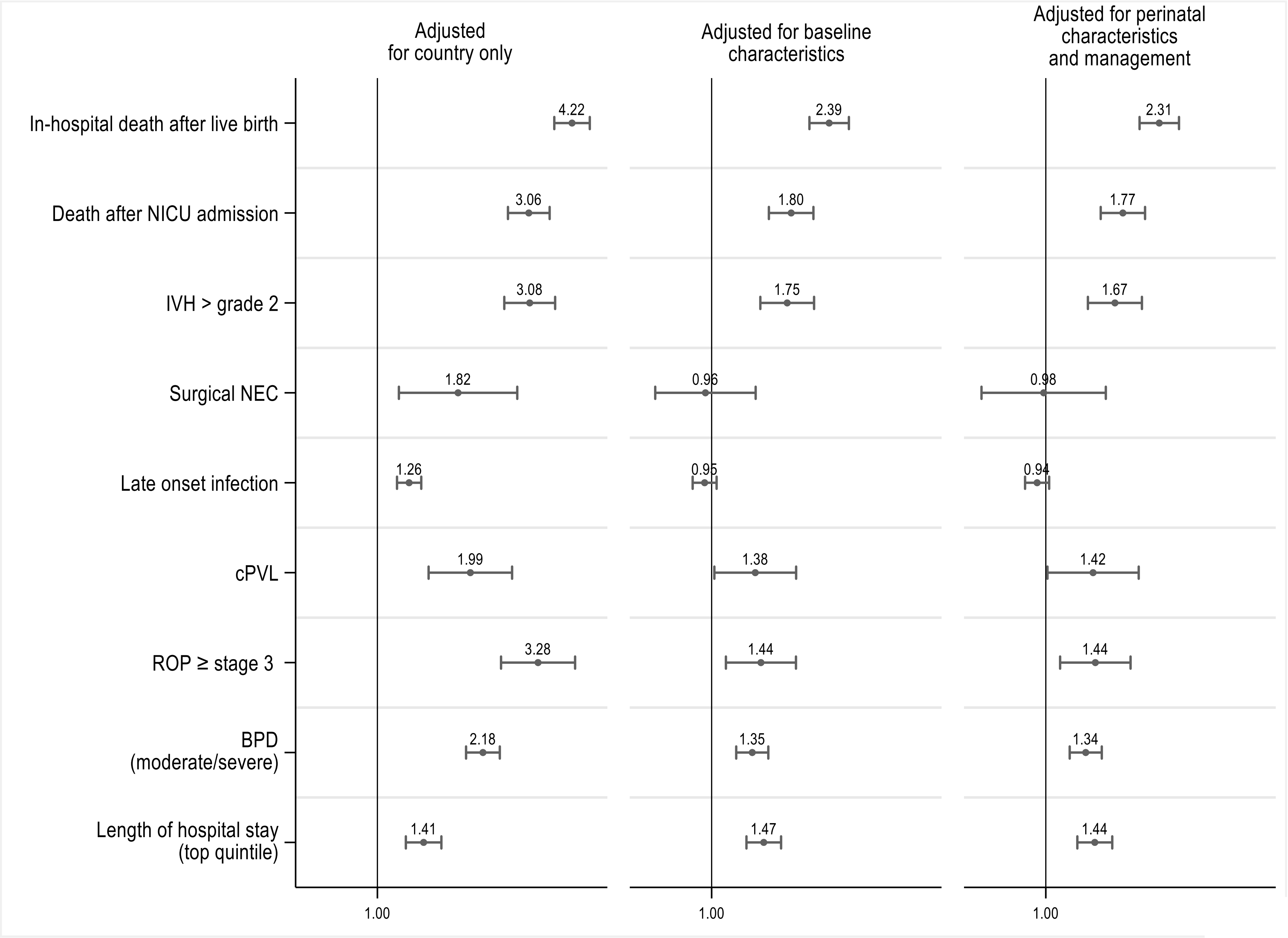
Adjusted for country only
Adjusted for baseline characteristics
Adjusted for perinatal characteristics and management
In-hospital death after live birth
Death after NICU admission
IVH > grade 2
Surgical NEC
Late onset infection
cPVL
ROP ≥ stage 3
BPD (moderate/severe)
Length of hospital stay (top quintile)
4.22
3.06
3.08
1.82
1.26
1.99
3.28
2.18
1.41
2.39
1.80
1.75
0.96
0.95
1.38
1.44
1.35
1.47
2.31
1.77
1.67
0.98
0.94
1.42
1.44
1.34
1.44
1.00
1.00
1.00

Figure S2

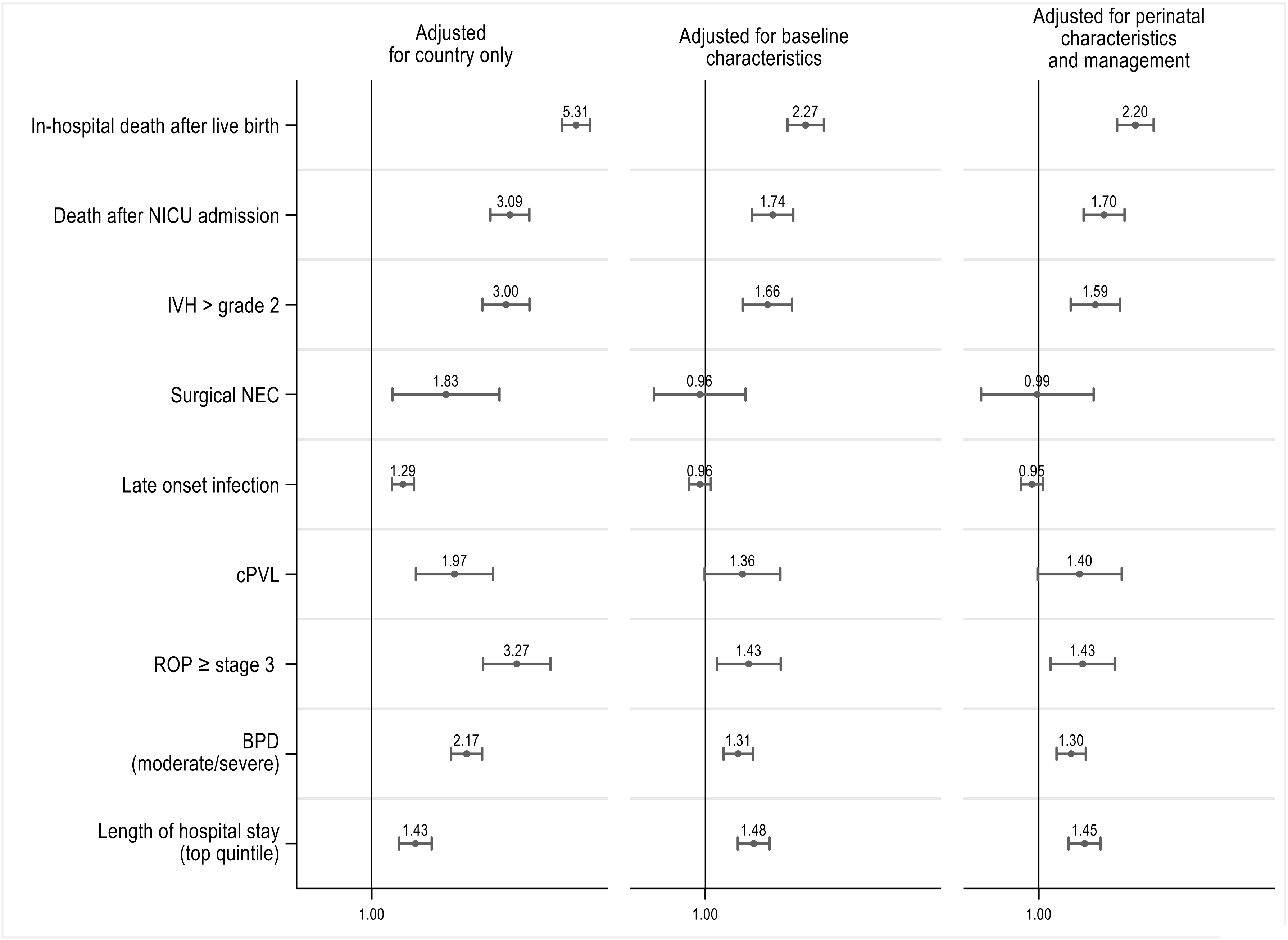
Adjusted
for country only
Adjusted for baseline
characteristics
Adjusted for perinatal
characteristics
and management
In-hospital death after live birth
Death after NICU admission
IVH > grade 2
Surgical NEC
Late onset infection
cPVL
ROP ≥ stage 3
BPD
(moderate/severe)
Length of hospital stay
(top quintile)
5.31
3.09
3.00
1.83
1.29
1.97
3.27
2.17
1.43
2.27
1.74
1.66
0.96
0.96
1.36
1.43
1.31
1.48
2.20
1.70
1.59
0.99
0.95
1.40
1.43
1.30
1.45
1.00
1.00
1.00

Figure S3

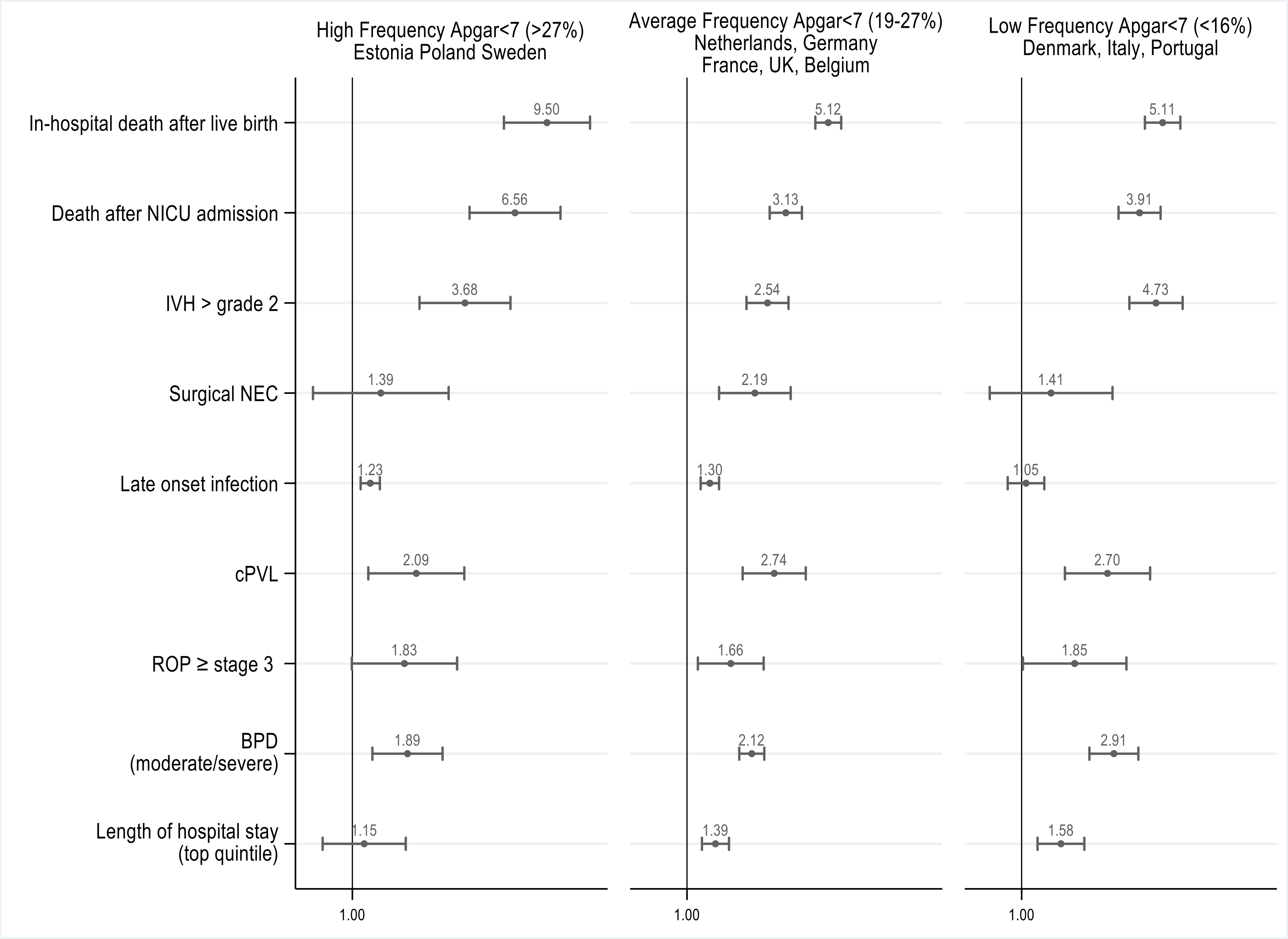

High Frequency Apgar<7 (>27%)
Estonia Poland Sweden
Average Frequency Apgar<7 (19-27%)
Netherlands, Germany
France, UK, Belgium
Low Frequency Apgar<7 (<16%)
Denmark, Italy, Portugal
In-hospital death after live birth
Death after NICU admission
IVH > grade 2
Surgical NEC
Late onset infection
cPVL
ROP ≥ stage 3
BPD (moderate/severe)
Length of hospital stay (top quintile)
9.50
6.56
3.68
1.39
1.23
2.09
1.83
1.89
1.15
5.12
3.13
2.54
2.19
1.30
2.74
1.66
2.12
1.39
5.11
3.91
4.73
1.41
1.05
2.70
1.85
2.91
1.58
1.00
1.00
1.00

Figure S4

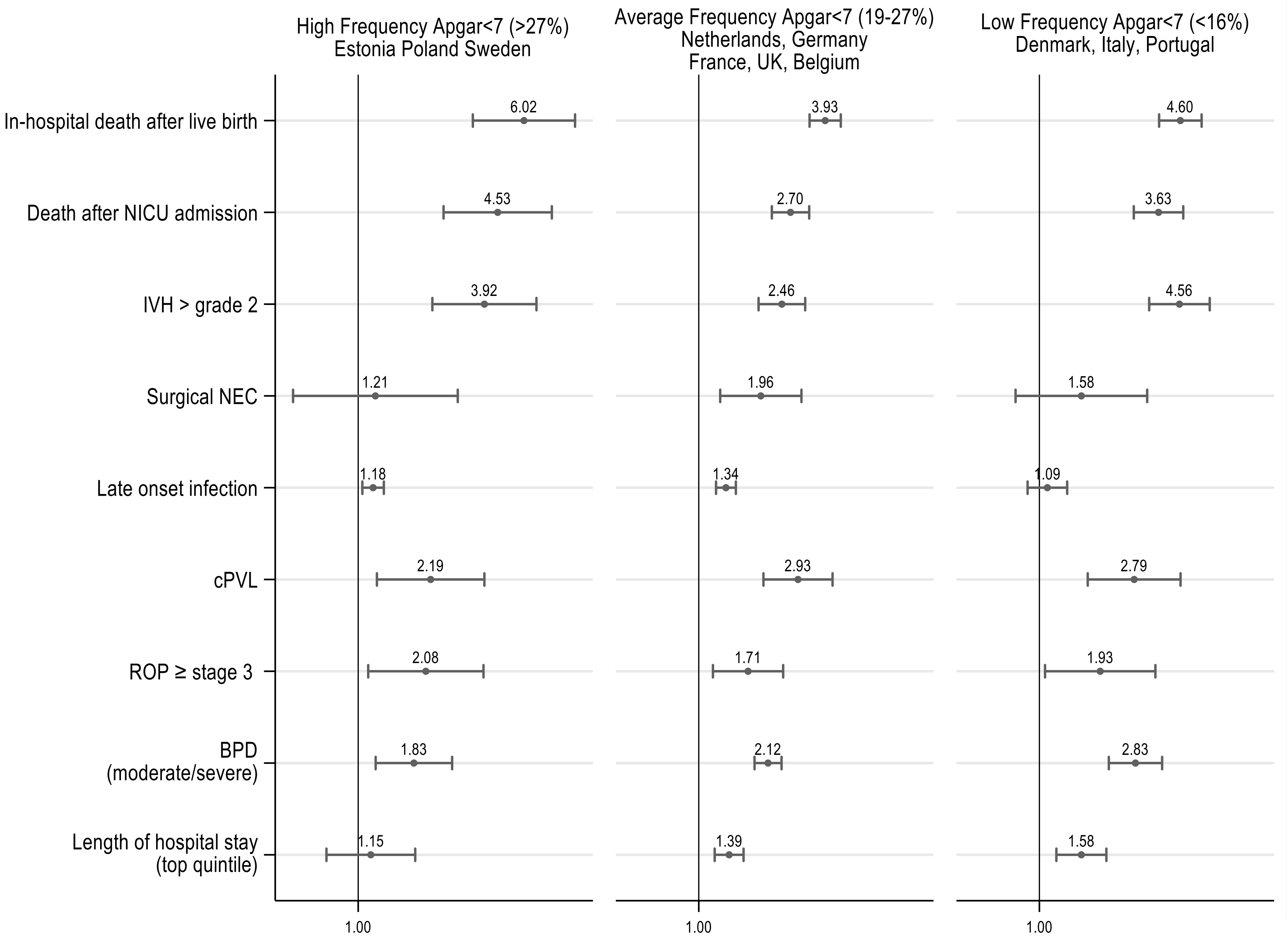
High Frequency Apgar<7 (>27%)
Estonia Poland Sweden
Average Frequency Apgar<7 (19-27%)
Netherlands, Germany
France, UK, Belgium
Low Frequency Apgar<7 (<16%)
Denmark, Italy, Portugal
In-hospital death after live birth
Death after NICU admission
IVH > grade 2
Surgical NEC
Late onset infection
cPVL
ROP ≥ stage 3
BPD
(moderate/severe)
Length of hospital stay
(top quintile)
6.02
4.53
3.92
1.21
1.18
2.19
2.08
1.83
1.15
3.93
2.70
2.46
1.96
1.34
2.93
1.71
2.12
1.39
4.60
3.63
4.56
1.58
1.09
2.79
1.93
2.83
1.58
1.00
1.00
1.00

Figure S5

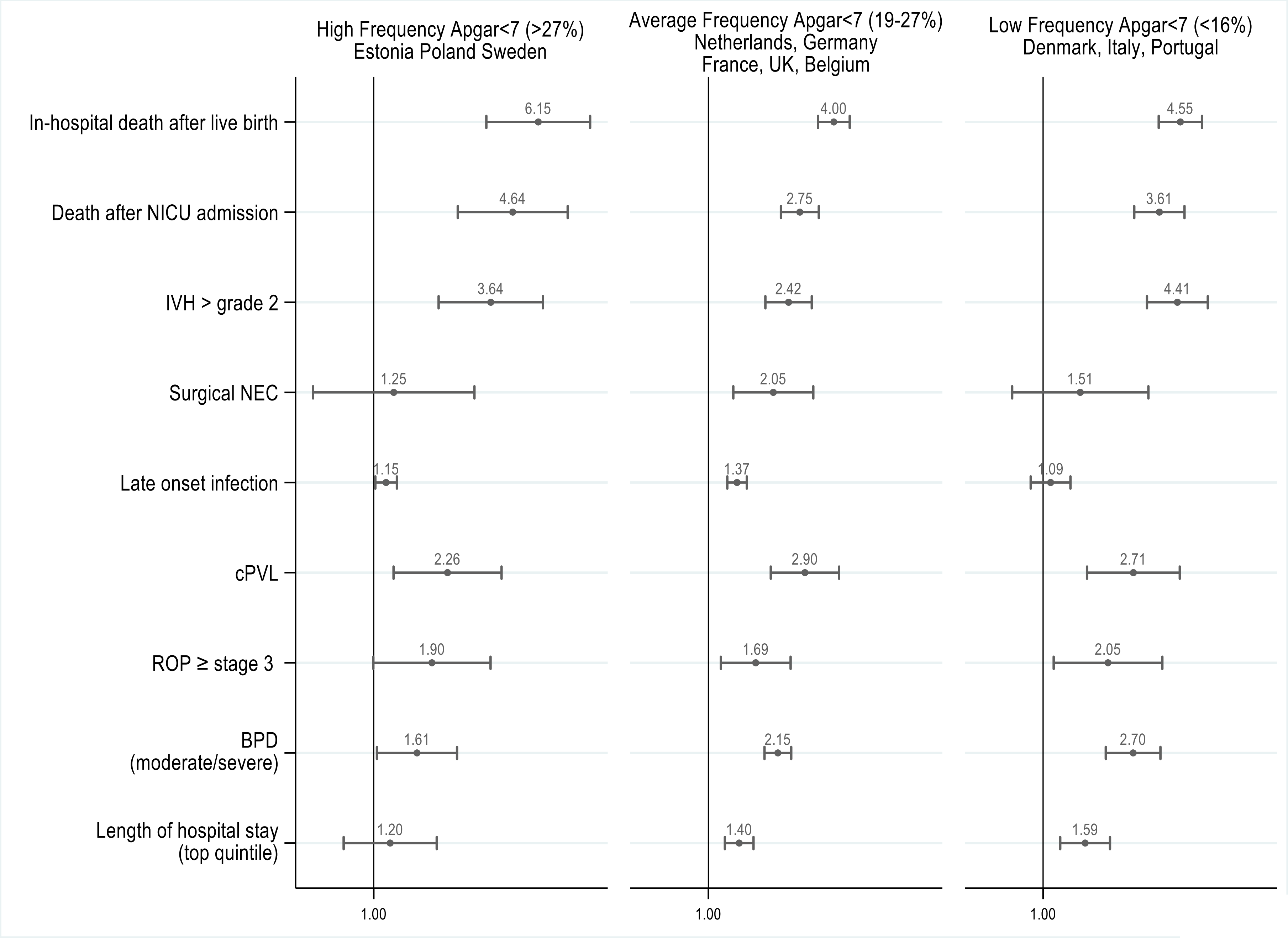
High Frequency Apgar<7 (>27%)
Estonia Poland Sweden
Average Frequency Apgar<7 (19-27%)
Netherlands, Germany
France, UK, Belgium
Low Frequency Apgar<7 (<16%)
Denmark, Italy, Portugal
In-hospital death after live birth
Death after NICU admission
IVH > grade 2
Surgical NEC
Late onset infection
cPVL
ROP ≥ stage 3
BPD
(moderate/severe)
Length of hospital stay
(top quintile)
6.15
4.64
3.64
1.25
1.15
2.26
1.90
1.61
1.20
4.00
2.75
2.42
2.05
1.37
2.90
1.69
2.15
1.40
4.55
3.61
4.41
1.51
1.09
2.71
2.05
2.70
1.59
1.00
1.00
1.00

Figure S6

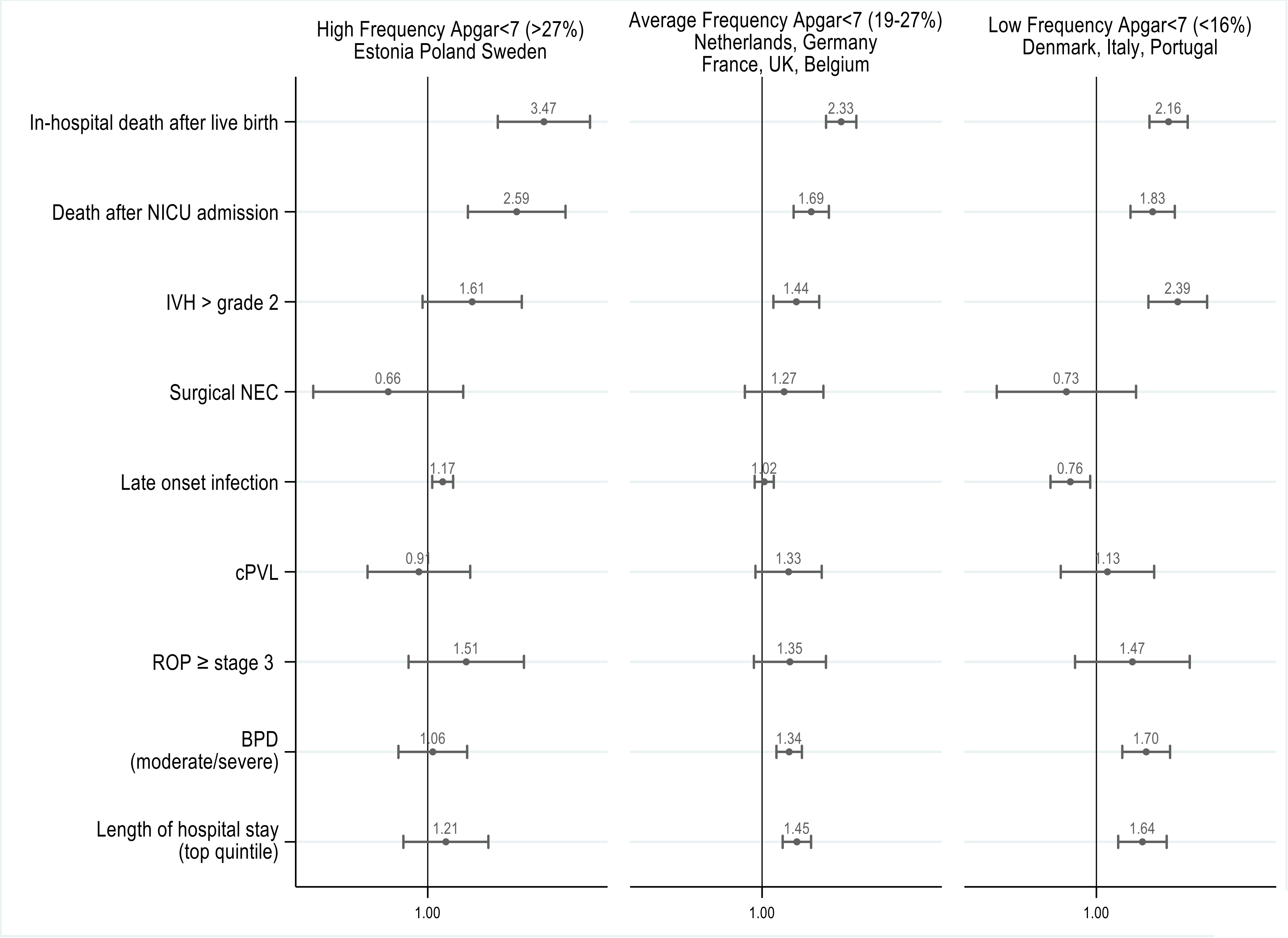
High Frequency Apgar<7 (>27%)
Estonia Poland Sweden
Average Frequency Apgar<7 (19-27%)
Netherlands, Germany
France, UK, Belgium
Low Frequency Apgar<7 (<16%)
Denmark, Italy, Portugal
In-hospital death after live birth
Death after NICU admission
IVH > grade 2
Surgical NEC
Late onset infection
cPVL
ROP ≥ stage 3
BPD
(moderate/severe)
Length of hospital stay
(top quintile)
3.47
2.59
1.61
0.66
1.17
0.91
1.51
1.06
1.21
2.33
1.69
1.44
1.27
1.02
1.33
1.35
1.34
1.45
2.16
1.83
2.39
0.73
0.76
1.13
1.47
1.70
1.64
1.00
1.00
1.00

Figure S7

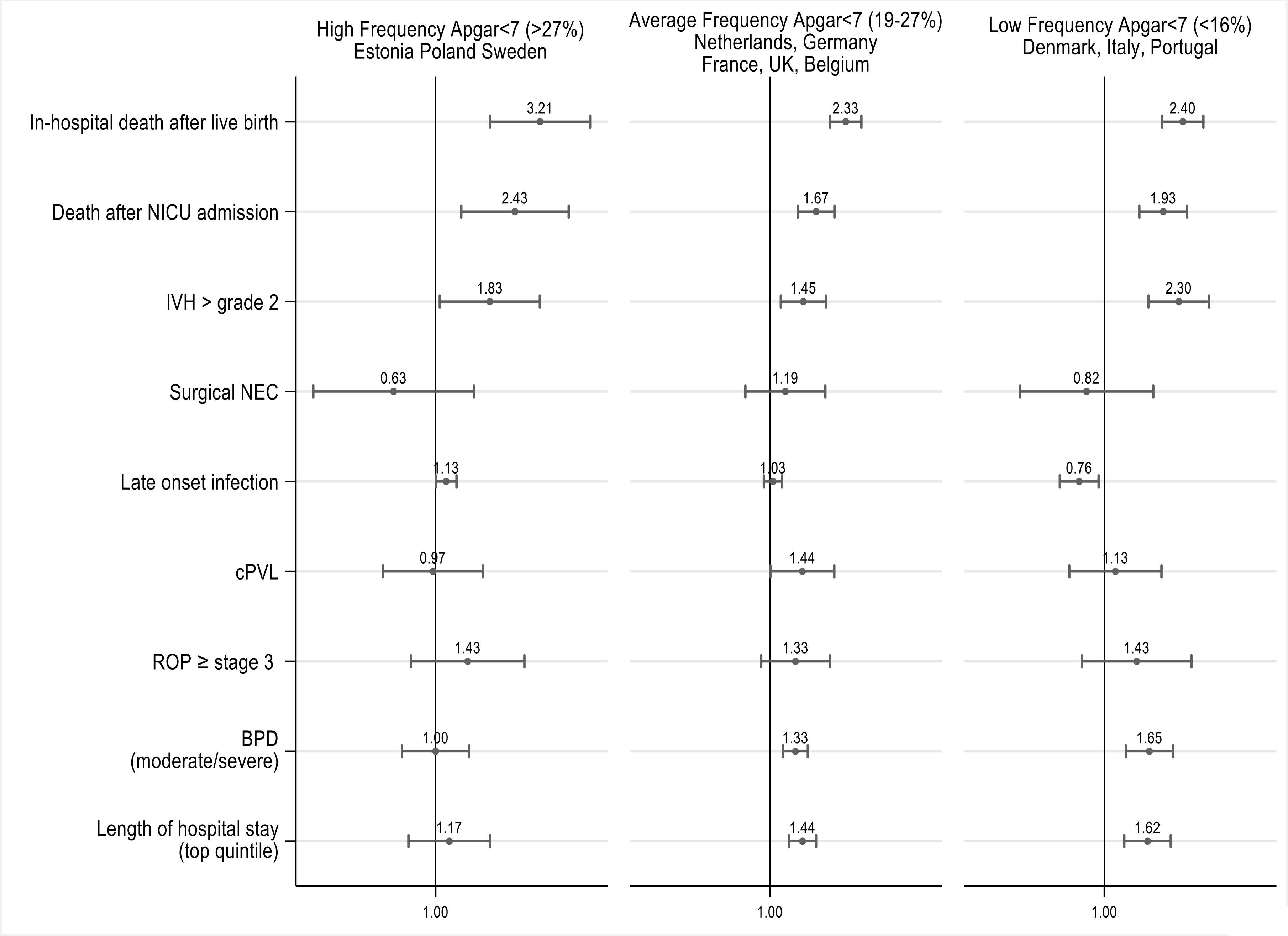
High Frequency Apgar<7 (>27%)
Estonia Poland Sweden
Average Frequency Apgar<7 (19-27%)
Netherlands, Germany
France, UK, Belgium
Low Frequency Apgar<7 (<16%)
Denmark, Italy, Portugal
In-hospital death after live birth
Death after NICU admission
IVH > grade 2
Surgical NEC
Late onset infection
cPVL
ROP ≥ stage 3
BPD
(moderate/severe)
Length of hospital stay
(top quintile)
3.21
2.43
1.83
0.63
1.13
0.97
1.43
1.00
1.17
2.33
1.67
1.45
1.19
1.03
1.44
1.33
1.33
1.44
2.40
1.93
2.30
0.82
0.76
1.13
1.43
1.65
1.62
1.00
1.00
1.00

Figure S8

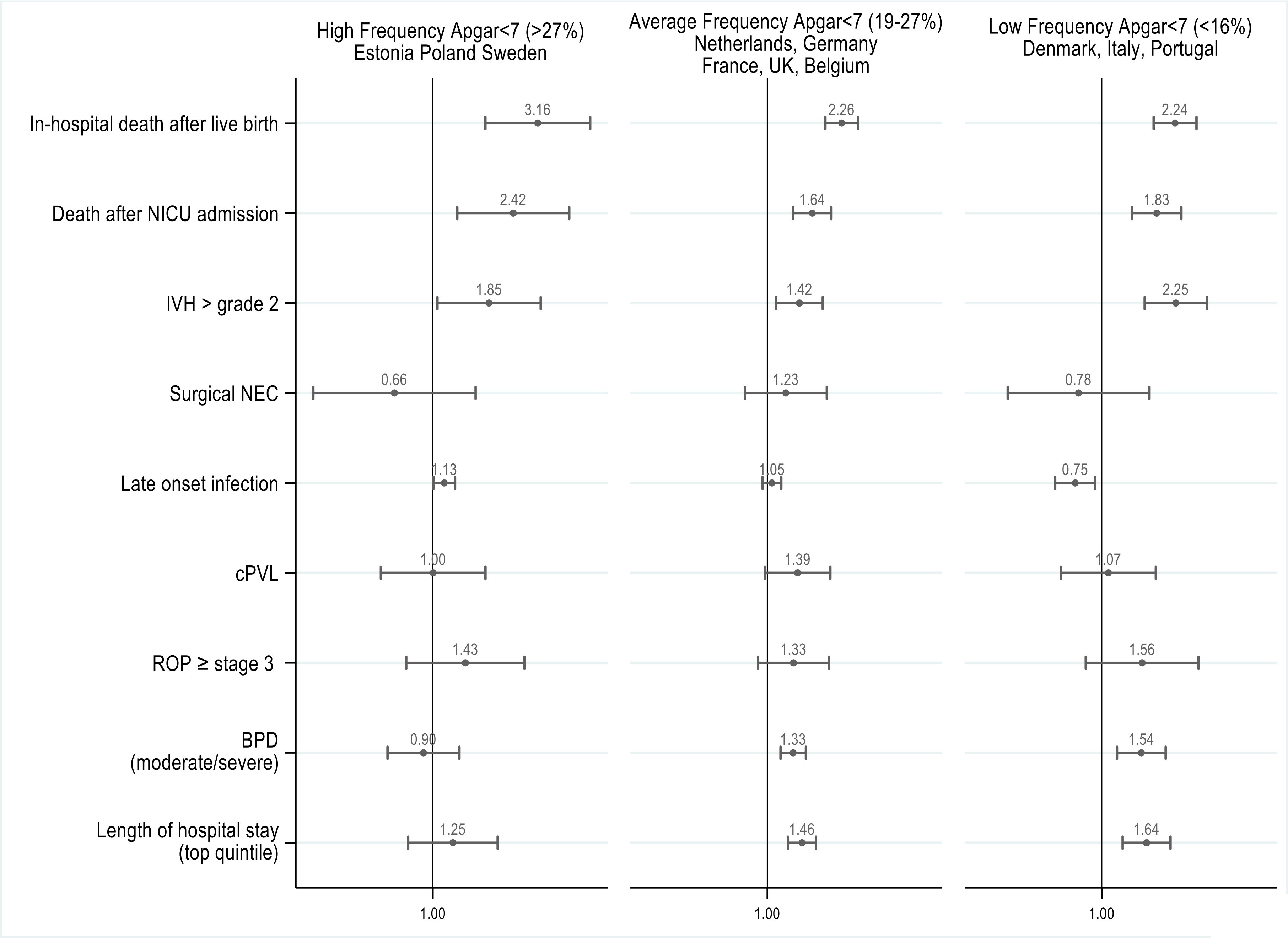
High Frequency Apgar<7 (>27%)
Estonia Poland Sweden
Average Frequency Apgar<7 (19-27%)
Netherlands, Germany
France, UK, Belgium
Low Frequency Apgar<7 (<16%)
Denmark, Italy, Portugal
In-hospital death after live birth
Death after NICU admission
IVH > grade 2
Surgical NEC
Late onset infection
cPVL
ROP ≥ stage 3
BPD
(moderate/severe)
Length of hospital stay
(top quintile)
3.16
2.42
1.85
0.66
1.13
1.00
1.43
0.90
1.25
2.26
1.64
1.42
1.23
1.05
1.39
1.33
1.33
1.46
2.24
1.83
2.25
0.78
0.75
1.07
1.56
1.54
1.64
1.00
1.00
1.00

Figure S9

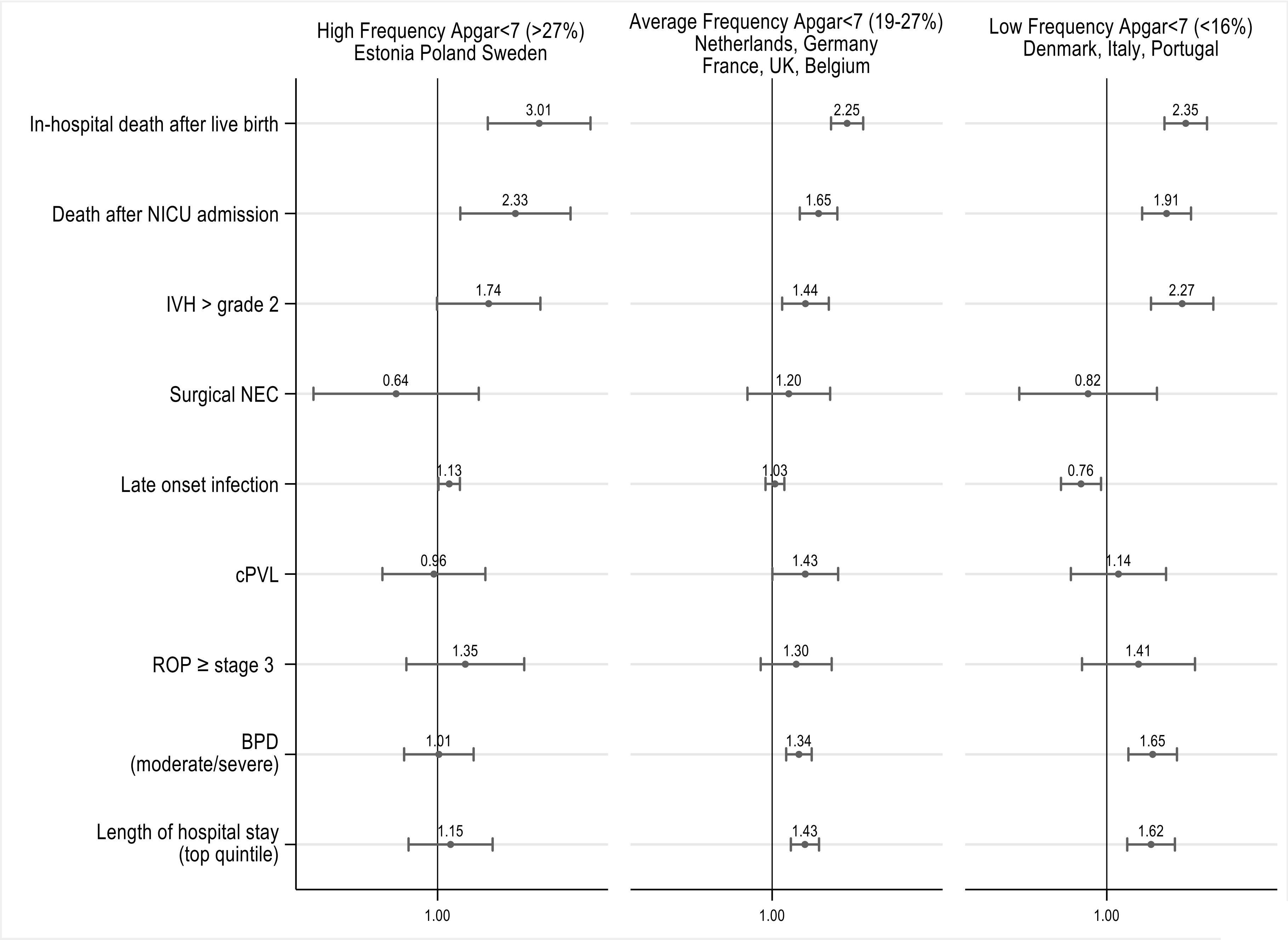

High Frequency Apgar<7 (>27%)
Estonia Poland Sweden
Average Frequency Apgar<7 (19-27%)
Netherlands, Germany
France, UK, Belgium
Low Frequency Apgar<7 (<16%)
Denmark, Italy, Portugal
In-hospital death after live birth
Death after NICU admission
IVH > grade 2
Surgical NEC
Late onset infection
cPVL
ROP ≥ stage 3
BPD
(moderate/severe)
Length of hospital stay
(top quintile)
3.01
2.33
1.74
0.64
1.13
0.96
1.35
1.01
1.15
2.25
1.65
1.44
1.20
1.03
1.43
1.30
1.34
1.43
2.35
1.91
2.27
0.82
0.76
1.14
1.41
1.65
1.62
1.00
1.00
1.00

Figure S10

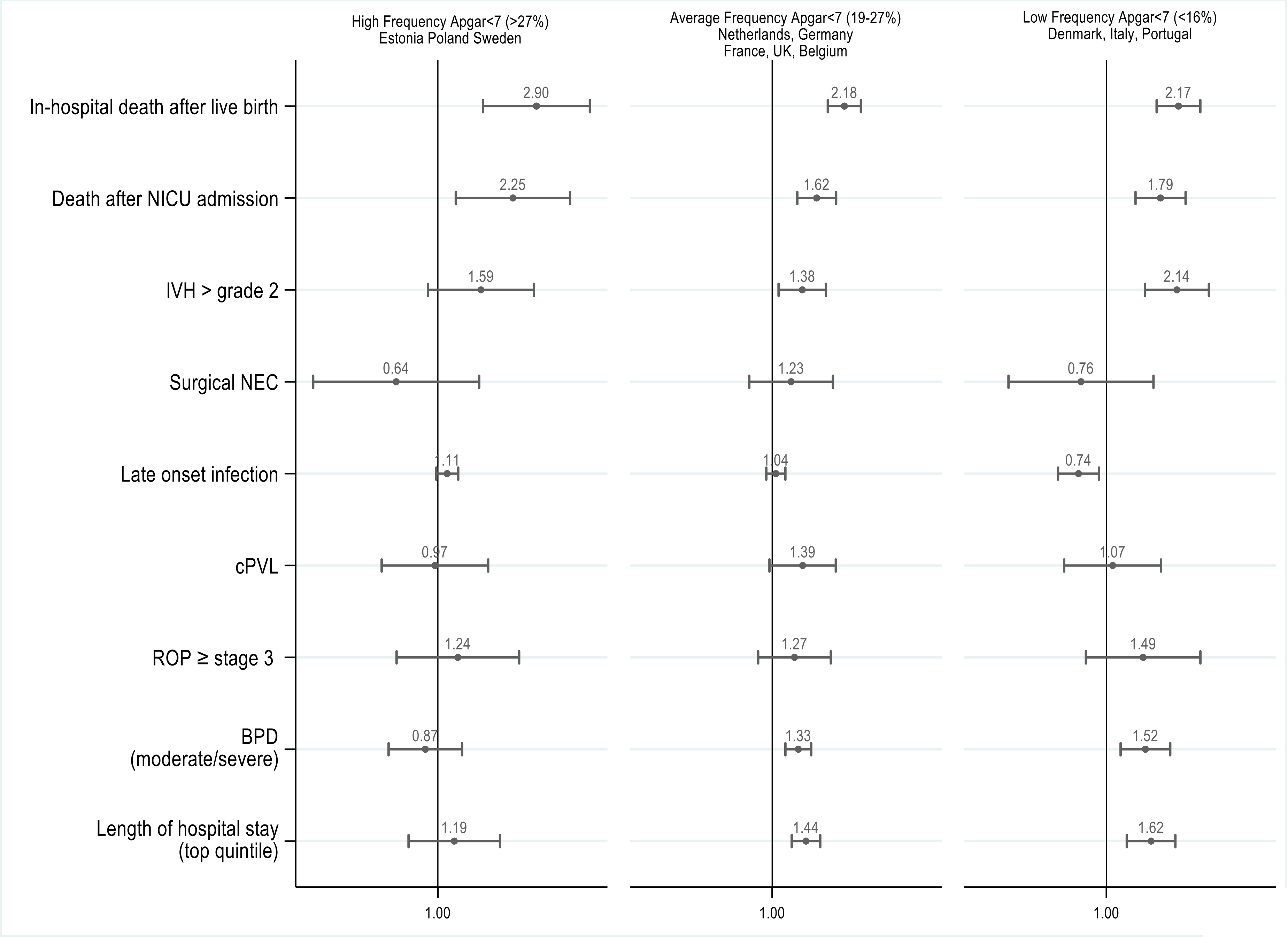
High Frequency Apgar<7 (>27%)
Estonia Poland Sweden
Average Frequency Apgar<7 (19-27%)
Netherlands, Germany
France, UK, Belgium
Low Frequency Apgar<7 (<16%)
Denmark, Italy, Portugal
In-hospital death after live birth
2.90
2.18
2.17
Death after NICU admission
2.25
1.62
1.79
IVH > grade 2
1.59
1.38
2.14
Surgical NEC
0.64
1.23
0.76
Late onset infection
1.11
1.04
0.74
cPVL
0.97
1.39
1.07
ROP ≥ stage 3
1.24
1.27
1.49
BPD
(moderate/severe)
0.87
1.33
1.52
Length of hospital stay
(top quintile)
1.19
1.44
1.62
1.00
1.00
1.00

Figure S11

Legends to Supplemental Figures

Figure S1. Relative risks of adverse neonatal outcome associated with an Apgar score <7 at five minutes in very preterm birth infants, all live births with complete case sample. Data from Figure 2A restricted to infants with complete case sample were analysed using a modified Poisson regression without adjustments except for country, with additional adjustment for baseline characteristics (gestational age, small for gestational age, multiple birth, sex, PPROM, parity, preeclampsia, eclampsia or HELLP syndrome), and plus adjustment for the perinatal management (antenatal steroids, inborn and composite variable of onset of labour and mode of delivery). Abbreviations: NICU, neonatal intensive care unit; IVH, intraventricular haemorrhage; NEC, necrotizing enterocolitis; cPVL, cystic periventricular leukomalacia; ROP, retinopathy of prematurity; BPD, bronchopulmonary dysplasia. Abbreviations: NICU, neonatal intensive care unit; IVH, intraventricular haemorrhage; NEC, necrotizing enterocolitis; cPVL, cystic periventricular leukomalacia; ROP, retinopathy of prematurity; BPD, bronchopulmonary dysplasia.

Figure S2. Relative risks of adverse neonatal outcome associated with an Apgar score <7 at five minutes in very preterm birth infants ≥ 24 weeks, all live births. Data from Figure 2A restricted to infants ≥24 weeks were analysed using a modified Poisson regression without adjustments except for country, with additional adjustment for baseline characteristics (gestational age, small for gestational age, multiple birth, sex, PPROM, parity, preeclampsia, eclampsia or HELLP syndrome), and plus adjustment for the perinatal management (antenatal steroids, inborn and composite variable of onset of labor and mode of delivery). Abbreviations: NICU, neonatal intensive care unit; IVH, intraventricular haemorrhage; NEC, necrotizing enterocolitis; cPVL, cystic periventricular leukomalacia; ROP, retinopathy of prematurity; BPD, bronchopulmonary dysplasia. Abbreviations: NICU, neonatal intensive care unit; IVH, intraventricular haemorrhage; NEC, necrotizing enterocolitis; cPVL, cystic periventricular leukomalacia; ROP, retinopathy of prematurity; BPD, bronchopulmonary dysplasia.

Figure S3. Relative risks of adverse neonatal outcome associated with an Apgar score <7 at five minutes in very preterm birth infants ≥ 24 weeks without severe congenital malformation, all live births. Data from Figure 2A restricted to infants ≥24 weeks without severe congenital malformation were analysed using a modified Poisson regression without adjustments except for country, with additional adjustment for baseline characteristics (gestational age, small for gestational age, multiple birth, sex, PPROM, parity, preeclampsia, eclampsia or HELLP syndrome), and plus adjustment for the perinatal management (antenatal steroids, inborn and composite variable of onset of labour and mode of delivery). Abbreviations: NICU, neonatal intensive care unit; IVH, intraventricular haemorrhage; NEC, necrotizing enterocolitis; cPVL, cystic periventricular leukomalacia; ROP, retinopathy of prematurity; BPD, bronchopulmonary dysplasia.

Figure S4. Unadjusted relative risks of adverse neonatal outcome among very preterm infants associated with an Apgar score <7 at five minutes by country group, classified by low Apgar prevalence. Analyses from Figure S2 were conducted using an interaction term to derive coefficients by country group: higher (Poland, Sweden, Estonia), average (the Netherlands, Germany, France, the United Kingdom, Belgium) and lower (Portugal, Italy, Denmark) frequencies of an Apgar score <7. Abbreviations: Avg., average; NICU, neonatal intensive care unit; IVH, intraventricular haemorrhage; NEC, necrotizing enterocolitis; cPVL, cystic periventricular leukomalacia; ROP, retinopathy of prematurity; BPD, bronchopulmonary dysplasia.

Figure S5. Unadjusted relative risks of adverse neonatal outcome among very preterm infants ≥ 24 weeks associated with an Apgar score <7 at five minutes by country group, classified by low Apgar prevalence. Analyses from Figure S2 restricted to very preterm infants ≥24 weeks were conducted using an interaction term to derive coefficients by country group: higher (Poland, Sweden, Estonia), average (the Netherlands, Germany, France, the UK, Belgium) and lower (Portugal, Italy, Denmark) frequencies of an Apgar score <7. Abbreviations: Avg., average; NICU, neonatal intensive care unit; IVH, intraventricular haemorrhage; NEC, necrotizing enterocolitis; cPVL, cystic periventricular leukomalacia; ROP, retinopathy of prematurity; BPD, bronchopulmonary dysplasia.

Figure S6. Unadjusted relative risks of adverse neonatal outcome among very preterm infants ≥ 24 weeks without severe congenital malformations associated with an Apgar score <7 at five minutes by country group, classified by low Apgar prevalence. Analyses from Figure S2 restricted to very preterm infants ≥24 weeks without severe congenital malformations were conducted using an interaction term to derive coefficients by country group: higher (Poland, Sweden, Estonia), average (the Netherlands, Germany, France, the UK, Belgium) and lower (Portugal, Italy, Denmark) frequencies of an Apgar score <7. Abbreviations: Avg., average; NICU, neonatal intensive care unit; IVH, intraventricular haemorrhage; NEC, necrotizing enterocolitis; cPVL, cystic periventricular leukomalacia; ROP, retinopathy of prematurity; BPD, bronchopulmonary dysplasia.

Figure S7. Relative risks of adverse neonatal outcome among very preterm infants associated with an Apgar score <7 at five minutes by country group, classified by low Apgar prevalence, adjusted for baseline characteristics. Analyses from Figure S2 adjusted for baseline characteristic were conducted using an interaction term to derive coefficients by country group: higher (Poland, Sweden, Estonia), average (the Netherlands, Germany, France, the UK, Belgium) and lower (Portugal, Italy, Denmark) frequencies of an Apgar score <7. Abbreviations: Avg., average; NICU, neonatal intensive care unit; IVH, intraventricular haemorrhage; NEC, necrotizing enterocolitis; cPVL, cystic periventricular leukomalacia; ROP, retinopathy of prematurity; BPD, bronchopulmonary dysplasia.

Figure S8. Relative risks of adverse neonatal outcome among very preterm infants ≥ 24 weeks associated with an Apgar score <7 at five minutes by country group, classified by low Apgar prevalence, adjusted for baseline characteristics. Analyses from Figure S2 restricted to very preterm infants ≥24 weeks adjusted for baseline characteristic were conducted using an interaction term to derive coefficients by country group: higher (Poland, Sweden, Estonia), average (the Netherlands, Germany, France, the UK, Belgium) and lower (Portugal, Italy, Denmark) frequencies of an Apgar score <7. Abbreviations: Avg., average; NICU, neonatal intensive care unit; IVH, intraventricular haemorrhage; NEC, necrotizing enterocolitis; cPVL, cystic periventricular leukomalacia; ROP, retinopathy of prematurity; BPD, bronchopulmonary dysplasia.

Figure S9. Relative risks of adverse neonatal outcome among very preterm infants ≥ 24 weeks without severe congenital malformations associated with an Apgar score <7 at five minutes by country group, classified by low Apgar prevalence, adjusted for baseline characteristics. Analyses from Figure S2 restricted to very preterm infants ≥24 weeks without severe congenital malformations adjusted for baseline characteristic were conducted using an interaction term to derive coefficients by country group: higher (Poland, Sweden, Estonia), average (the Netherlands, Germany, France, the UK, Belgium) and lower (Portugal, Italy, Denmark) frequencies of an Apgar score <7. Abbreviations: Avg., average; NICU, neonatal intensive care unit; IVH, intraventricular haemorrhage; NEC, necrotizing enterocolitis; cPVL, cystic periventricular leukomalacia; ROP, retinopathy of prematurity; BPD, bronchopulmonary dysplasia.

Figure S10. Relative risks of adverse neonatal outcome among very preterm infants ≥ 24 weeks associated with an Apgar score <7 at five minutes by country group, classified by low Apgar prevalence, adjusted for baseline characteristics and perinatal management. Analyses from Figure S2 restricted to very preterm infants ≥24 weeks adjusted for baseline characteristic and perinatal management were conducted using an interaction term to derive coefficients by country group: higher (Poland, Sweden, Estonia), average (the Netherlands, Germany, France, the UK, Belgium) and lower (Portugal, Italy, Denmark) frequencies of an Apgar score <7. Abbreviations: Avg., average; NICU, neonatal intensive care unit; IVH, intraventricular haemorrhage; NEC, necrotizing enterocolitis; cPVL, cystic periventricular leukomalacia; ROP, retinopathy of prematurity; BPD, bronchopulmonary dysplasia.

Figure S11. Relative risks of adverse neonatal outcome among very preterm infants ≥ 24 weeks without severe congenital malformations associated with an Apgar score <7 at five minutes by country group, classified by low Apgar prevalence, adjusted for baseline characteristics and perinatal management. Analyses from Figure S2 restricted to very preterm infants ≥24 weeks without severe congenital malformations adjusted for baseline characteristic and perinatal management were conducted using an interaction term to derive coefficients by country group: higher (Poland, Sweden, Estonia), average (the Netherlands, Germany, France, the UK, Belgium) and lower (Portugal, Italy, Denmark) frequencies of an Apgar score <7. Abbreviations: Avg., average; NICU, neonatal intensive care unit; IVH, intraventricular haemorrhage; NEC, necrotizing enterocolitis; cPVL, cystic periventricular leukomalacia; ROP, retinopathy of prematurity; BPD, bronchopulmonary dysplasia.

Supplemental Tables

Table S1. Maternal and perinatal characteristics of infants with and without Apgar scores.

	All births		Available Apgar score N=7396		Missing Apgar score N=504	
	N	%	N	%	N	%
Maternal age at childbirth, years						
<25	1,345	17.1%	1,250	17.0%	95	19.3%
25-34	4,450	56.6%	4,192	56.9%	258	52.3%
>=35	2,067	26.3%	1,927	26.2%	140	28.4%
Maternal parity						
Primiparous	4,392	56.3%	4,125	56.3%	267	55.9%
Multiparous	3,415	43.7%	3,204	43.7%	211	44.1%
Country of birth						
Born in country	5,733	76.1%	5,382	75.9%	351	78.9%
Born in another European country	480	6.4%	443	6.2%	37	8.3%
Born outside of Europe	1,323	17.6%	1,266	17.9%	57	12.8%
Continental country classification						
Africa	794	10.5%	753	10.6%	41	9.2%
America	137	1.8%	135	1.9%	2	0.4%
Asia[1]	392	5.2%	378	5.3%	14	3.1%
Europe	6,213	82.4%	5,825	82.1%	388	87.2%
Type of pregnancy						
Singleton	5,437	68.8%	5,083	68.7%	354	70.4%
Multiple	2,461	31.2%	2,312	31.3%	149	29.6%
Preeclampsia, eclampsia or HELLP syndrome						
No	6,573	85.1%	6,151	84.7%	422	90.8%
Yes	1,153	14.9%	1,110	15.3%	43	9.2%
PPROM >12h						
No	5,790	75.0%	5,451	75.1%	339	73.5%
Yes	1,931	25.0%	1,809	24.9%	122	26.5%
Inborn						
No	860	11.3%	769	10.7%	91	20.9%
Yes	6,761	88.7%	6,417	89.3%	344	79.1%
Any antenatal steroids						
No	1,055	13.5%	927	12.6%	128	26.6%

Yes	6,759	86.5%	6,405	87.4%	354	73.4%
Mode of delivery						
Vaginal birth	2,494	32.0%	2,269	31.0%	225	46.8%
Instrumental vaginal birth	185	2.4%	170	2.3%	15	3.1%
Prelabour caesarean	3,193	40.9%	3,037	41.5%	156	32.4%
Intrapartum caesarean	1,927	24.7%	1,842	25.2%	85	17.7%
Sex of the infant						
Male	4,266	54.0%	4,012	54.3%	254	50.5%
Female	3,627	45.9%	3,380	45.7%	247	49.1%
Undetermined	3	0.0%	1	0.0%	2	0.4%
Gestational age, completed weeks						
23/24	717	9.1%	604	8.2%	113	22.4%
25	448	5.7%	400	5.4%	48	9.5%
26	604	7.6%	548	7.4%	56	11.1%
27	753	9.5%	697	9.4%	56	11.1%
28	926	11.7%	868	11.7%	58	11.5%
29	1,053	13.3%	1,000	13.5%	53	10.5%
30	1,486	18.8%	1,439	19.5%	47	9.3%
31	1,913	24.2%	1,840	24.9%	73	14.5%
SGA centiles, using intrauterine charts						
<3	1,633	20.7%	1,535	20.8%	98	19.6%
3-10	896	11.4%	844	11.4%	52	10.4%
>10	5,363	68.0%	5,013	67.8%	350	70.0%
Severe congenital malformation						
No	7,773	98.4%	7,280	98.4%	493	97.8%
Yes	126	1.6%	115	1.6%	11	2.2%

Data are given as absolute numbers and percentages of rows separated by the Apgar score. Total number of patients differs for variations ins missing values for individual covariables. Abbreviations: PPROM, preterm premature rupture of membranes; C-section, caesarean section; SGA, small for gestational age; ANS, antenatal steroids. [1]Includes 2 women from the Pacific islands.

Table S2: Characteristics of infants in the three country Apgar groups

	Low<br>N = 2032		Medium<br>N = 4656		High<br>N = 719	
	N	%	N	%	N	%
Maternal age at childbirth, years						
<25	273	12.4%	953	19.3%	119	16.3%
25-34	1,135	51.5%	2,913	59.1%	402	55.0%
>=35	797	36.1%	1,060	21.5%	210	28.7%
Maternal parity						
Primiparous	1,340	61.3%	2,693	55.1%	359	49.0%
Multiparous	846	38.7%	2,195	44.9%	374	51.0%
Country of birth						
Born in country	1,597	73.4%	3,512	75.9%	624	85.0%
Born in another European country	249	11.4%	199	4.3%	32	4.4%
Born outside of Europe	331	15.2%	914	19.8%	78	10.6%
Continental country classification						
Africa	153	7.0%	607	13.1%	34	4.6%
America	88	4.0%	32	0.7%	15	2.0%
Asia[1]	90	4.1%	275	5.9%	29	3.8%
Europe	1,846	84.8%	3,711	80.2%	656	89.4%
Type of pregnancy						
Singleton	1,476	66.8%	3,406	68.8%	555	75.4%
Multiple	733	33.2%	1,547	31.2%	181	24.6%
Preeclampsia, eclampsia or HELLP syndrome						
No	1,880	85.6%	4,066	84.7%	627	86.4%
Yes	317	14.4%	737	15.3%	99	13.6%
PPROM >12h						
No	1,697	77.3%	3,572	74.4%	521	71.9%
Yes	497	22.7%	1,230	25.6%	204	28.1%
Inborn						
No	185	8.6%	535	11.3%	140	19.6%
Yes	1,977	91.4%	4,208	88.7%	576	80.4%
Any antenatal steroids						
No	196	8.9%	744	15.2%	115	15.7%
Yes	1,998	91.1%	4,142	84.8%	619	84.3%
Mode of delivery						
Vaginal birth	535	24.3%	1,698	34.9%	261	35.6%

Instrumental vaginal birth	15	0.7%	169	3.5%	1	0.1%
Prelabour caesarean	1,069	48.5%	1,891	38.9%	233	31.7%
Intrapartum caesarean	585	26.5%	1,103	22.7%	239	32.6%
Sex of the infant						
Male	1,171	53.0%	2,694	54.4%	401	54.5%
Female	1,038	47.0%	2,254	45.5%	335	45.5%
Undetermined	0	0.0%	3	0.1%	0	0.0%
Gestational age completed weeks						
23/24	181	8.2%	448	9.0%	88	12.0%
25	116	5.3%	284	5.7%	48	6.5%
26	174	7.9%	385	7.8%	45	6.1%
27	257	11.6%	430	8.7%	66	9.0%
28	269	12.2%	574	11.6%	83	11.3%
29	280	12.7%	677	13.7%	96	13.0%
30	393	17.8%	955	19.3%	138	18.8%
31	539	24.4%	1,202	24.3%	172	23.4%
SGA centiles, using intrauterine charts						
<3	472	21.4%	1,032	20.9%	129	17.5%
3-10	255	11.5%	551	11.1%	90	12.2%
>10	1,481	67.1%	3,365	68.0%	517	70.2%
Severe congenital malformation						
No	2,178	98.6%	4,882	98.5%	713	96.9%
Yes	31	1.4%	72	1.5%	23	3.1%

Data are given as absolute numbers and percentages of rows separated by the country Apgar group. Total number of patients differs for variations ins missing values for individual covariables. Abbreviations: PPROM, preterm premature rupture of membranes; C-section, caesarean section; SGA, small for gestational age; ANS, antenatal steroids. [1]Includes 2 women from the Pacific islands.
